# Assessing the relationship between gut microbiota and endometriosis: a bidirectional two-sample mendelian randomization analysis

**DOI:** 10.1186/s12905-024-02945-z

**Published:** 2024-02-16

**Authors:** Chunxiao Dang, Zhenting Chen, Yuyan Chai, Pengfei Liu, Xiao Yu, Yan Liu, Jinxing Liu

**Affiliations:** 1 First Clinical Medical College, Shandong University of Traditional Chinese Medicine, Jinan, 250355 Shandong China; 2 Department of eugenic genetics, Dongying People’s Hospital (Dongying Hospital of Shandong Provincial Hospital Group), Dongying, 257091 Shandong China; 3 Department of obstetrics, The People’s Hospital of Dongying Distric, Dongying, 257091 Shandong China; 4 Department of gynaecology, Affiliated Hospital of Shandong University of Traditional Chinese Medicine, Jinan, 250000 Shandong China; 5 National Key Laboratory for Innovation and Transformation of Luobing Theory, The Key Laboratory of Cardiovascular Remodeling and Function Research, Department of Cardiology, Chinese Ministry of Education, Chinese National Health Commission and Chinese Academy of Medical Sciences, Qilu Hospital of Shandong University, Jinan, 250000 Shandong China

**Keywords:** Mendelian randomization study, Gut microbiota, Endometriosis, Causal effects

## Abstract

**Background:**

An increasing body of observational studies have indicated an association between gut microbiota and endometriosis. However, the causal relationship between them is not yet clear. In this study, we employed Mendelian randomization method to investigate the causal relationship between 211 gut microbiota taxa and endometriosis.

**Methods:**

Independent genetic loci significantly associated with the relative abundance of 211 gut microbiota taxa, based on predefined thresholds, were extracted as instrumental variables. The primary analytical approach employed was the IVW method. Effect estimates were assessed primarily using the odds ratio and 95% confidence intervals. Supplementary analyses were conducted using MR-Egger regression, the weighted median method, the simple mode and the weighted mode method to complement the IVW results. In addition, we conducted tests for heterogeneity, horizontal pleiotropy, sensitivity analysis, and MR Steiger to assess the robustness of the results and the strength of the causal relationships.

**Results:**

Based on the IVW method, we found that the *family Prevotellaceae*, *genus Anaerotruncus*, *genus Olsenella*, *genus Oscillospira*, and *order Bacillales* were identified as risk factors for endometriosis, while *class Melainabacteria* and *genus Eubacterium ruminantium group* were protective factors. Additionally, no causal relationship was observed between endometriosis and gut microbiota. Heterogeneity tests, pleiotropy tests, and leave-one-out sensitivity analyses did not detect any significant heterogeneity or pleiotropic effects.

**Conclusions:**

Our MR study has provided evidence supporting a potential causal relationship between gut microbiota and endometriosis, and it suggests the absence of bidirectional causal effects. These findings could potentially offer new insights for the development of novel strategies for the prevention and treatment of endometriosis.

**Supplementary Information:**

The online version contains supplementary material available at 10.1186/s12905-024-02945-z.

## Introduction

Endometriosis (EMs) is a chronic, estrogen-dependent inflammatory condition characterized by the presence of endometrial tissue outside the uterus [1]. Approximately 6–10% of women of reproductive age are affected by EMs, and about 50% of infertile women have EMs [2, 3]. Due to the secretive and diverse nature of EMs symptoms, and the lack of reliable non-invasive methods for detecting endometriosis, it often goes unnoticed. In recent years, the gut microbiota has emerged as a research hotspot, with scholars [4–6] discovering its associations with various diseases such as gastrointestinal disorders, cardiovascular diseases, respiratory diseases, and more. Research on the relationship between gut microbiota and endometriosis has spanned over two decades, starting as early as the 1990s and continuing to the present day. Many scholars have observed significant differences in the types, distribution, and abundance of gut microbiota between patients with EMs and healthy women [7, 8]. Additionally, up to 90% of EMs patients experience gastrointestinal issues such as nausea, vomiting, diarrhea, and bloating [9], suggesting a potential imbalance in the gut microbiota. In fact, in a large-scale study, EMs patients were found to have a 50% increased risk of developing inflammatory bowel disease (IBD) compared to the general population [10]. Furthermore, ecological imbalances in the gut, vagina, or uterus in EMs patients may impact estrogen metabolism, immune system balance, and exacerbate the condition [11, 12]. However, in observational studies, the relationship between gut microbiota and endometriosis can be influenced by confounding factors (such as age and surgical history) and reverse causality, making it uncertain whether these associations are causal in nature.

Randomized controlled trials (RCTs) are considered the gold standard in epidemiology for inferring causal relationships. However, due to ethical constraints, implementing RCTs can be challenging [13]. Mendelian randomization (MR) utilizes single nucleotide polymorphism (SNP) loci as instrumental variables to infer causal associations between exposures and outcomes. It does so by adhering to the genetic principle of “random allocation of parental alleles to offspring,” achieving similar randomization effects without being influenced by external environmental factors, thus compensating for the limitations of observational studies [14].

Currently, there are no MR reports regarding a causal relationship between gut microbiota and endometriosis. Although previous observational studies have suggested an association between gut microbiota and the incidence and progression of endometriosis, the causal relationship is not yet clear. This study is the first application of a two-sample Mendelian randomization approach to explore the causal association between gut microbiota and endometriosis. It aims to provide new insights into the treatment and prevention of endometriosis.

## Materials and methods

### Research design

**Fig. 1:**
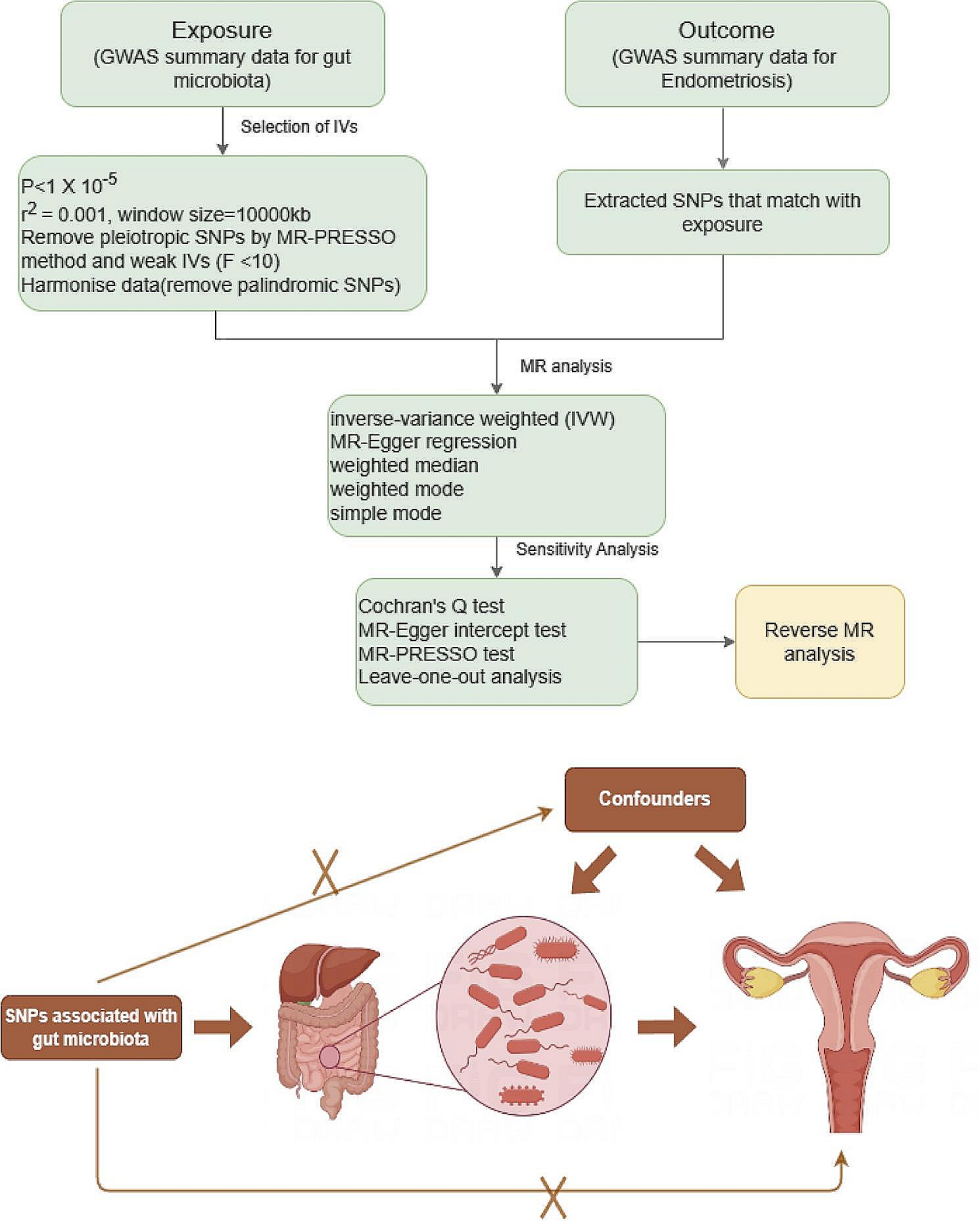
Flowchart of instrumental variable screening for MR method analysis

In a scenario where the genome wide association study (GWAS) summary data for the exposure variable and the GWAS summary data for the outcome variable are mutually independent, this study employed the TwoSampleMR package in R programming language to conduct a two-sample bidirectional Mendelian randomization analysis. The objective was to investigate the causal association between gut microbiota and endometriosis, with the specific design as shown in Fig. 1. MR analysis adheres to three crucial assumptions [15]: First, the instrumental variables are strongly correlated with the exposure variable. Second, the instrumental variables are independent of observed or unobserved confounding factors. Third, the instrumental variables affect the outcome solely through the exposure.

### Data source

The GWAS summary data for endometriosis were obtained from the Finngen database, which includes data from 77,257 European participants and covers 16,377,306 SNPs (https://gwas.mrcieu.ac.uk/datasets/finn-b-N14_ENDOMETRIOSIS/). The statistical data on gut microbiota were derived from the research conducted by the MiBioGen Consortium (http://www.mibiogen.org/), which incorporated 18,340 individuals from 24 cohorts, mainly from Europe [16]. Microbial composition was analyzed using three distinct variable regions of the targeted 16 S rRNA gene, namely V4 (10,413 samples, 13 cohorts), V3-V4 (4,211 samples, 6 cohorts), and V1-V2 (3,716 samples, 5 cohorts). Supplementary File 1 shows a description of the participants in each cohort in a dataset of gut microbiota. Both gut microbiota and endometriosis were selected as exposure and outcome variables, respectively, for the MR analysis. As our study is based on publicly available databases, ethical committee approval was not required.

### Instrumental variable selection

(1) IVs Selection: To obtain strongly related exposure data, SNPs with a significance level of *P* < 5 × 10^− 8^ were selected as conditions. Given that gut microbiota SNPs rarely have *P* < 5 × 10^− 8^, gut microbiota SNPs were selected with a threshold of *P* < 1 × 10^− 5^. (2) Independence Criterion: The PLINK aggregation method was used to calculate linkage disequilibrium (LD) between each risk factor’s SNPs. SNPs with an LD coefficient r^2^ > 0.001 and a physical distance of less than 10,000 kb were removed to ensure that the SNPs were mutually independent and to eliminate the influence of genetic pleiotropy on the results [17, 18]. (3) Statistical Strength Criteria: The strength of the instrumental variables was calculated using the F-statistic, with the formula: F = β^2^ / SE^2^ (where β is the allele effect size and SE is the standard error). Instrumental variables with F < 10 were removed to ensure that the instrumental variables were unrelated to unmeasured confounding factors [19]. Finally, the “harmonise_data” function from the TwoSampleMR package was used to align the direction of alleles between exposure and outcome, remove palindromic and incompatible SNPs [20], and exclude SNPs with confounding factors through the PhenoScanner database (http://www.phenoscanner.medschl.cam.ac.uk/).

### Mendelian randomization analysis

In this study, the inverse variance weighted (IVW) method [21] was employed as the primary analytical approach for establishing causal relationships. This method, assuming the validity of all instrumental variables, calculates weighted estimates by taking the reciprocal of their variances as weights. It provides the most accurate results when there is no heterogeneity or horizontal pleiotropy present. Additionally, MR-Egger regression, the weighted median (WME) method, the simple mode (SM) and the weighted mode (WM) method were used as supplementary analyses to complement the IVW results. MR-Egger regression method performs weighted linear regression of the exposure and outcome effect estimates, providing a causal effect assessment even when all SNPs are invalid instruments. The WME method leverages the intermediate effects of all available genetic variations, estimating them by weighting each SNP by the inverse variance of its correlation with the outcome. SM and WM are mode-based methods. The mode-based estimation model clusters SNPs with similar causal effects and returns causal effect estimates for the majority of clustered SNPs. Specifically, WM weights the influence of each SNP on the cluster by the inverse variance of its outcome effect. These methods complement the IVW results and provide additional insights into the causal relationships between exposure and outcome variables. Finally, we conducted reverse MR analysis for EMs and gut microbiota. The methods and settings used in these reverse MR analysis were consistent with those of forward MR.

### Sensitivity analysis

Heterogeneity testing [22] assesses the presence of differences among various IVs. It utilizes the *P*-value from Cochran’s Q test to evaluate heterogeneity, with *P* > 0.05 indicating the absence of heterogeneity. If heterogeneity is detected, the MR pleiotropy residual sum and outlier (MR-PRESSO) test is employed to assess potential outliers [23], eliminate them, and then reanalyze the data. Multiplicity testing [24] verifies the reliability of MR analysis results. MR-Egger intercept is used to detect horizontal pleiotropy, with *P* > 0.05 indicating the absence of horizontal pleiotropy and, thus, the reliability of the MR analysis results. Sensitivity testing [25] is conducted using a “leave-one-out” approach, sequentially removing each SNP. If the MR results derived from the remaining SNPs do not exhibit significant differences from the overall result, it demonstrates the robustness of the MR results. Furthermore, the MR Steiger directional test was employed to further assess the correlation between the exposure and the outcome.

## Results

### Causal effect of gut microbiota on EMs

**Fig. 2:**
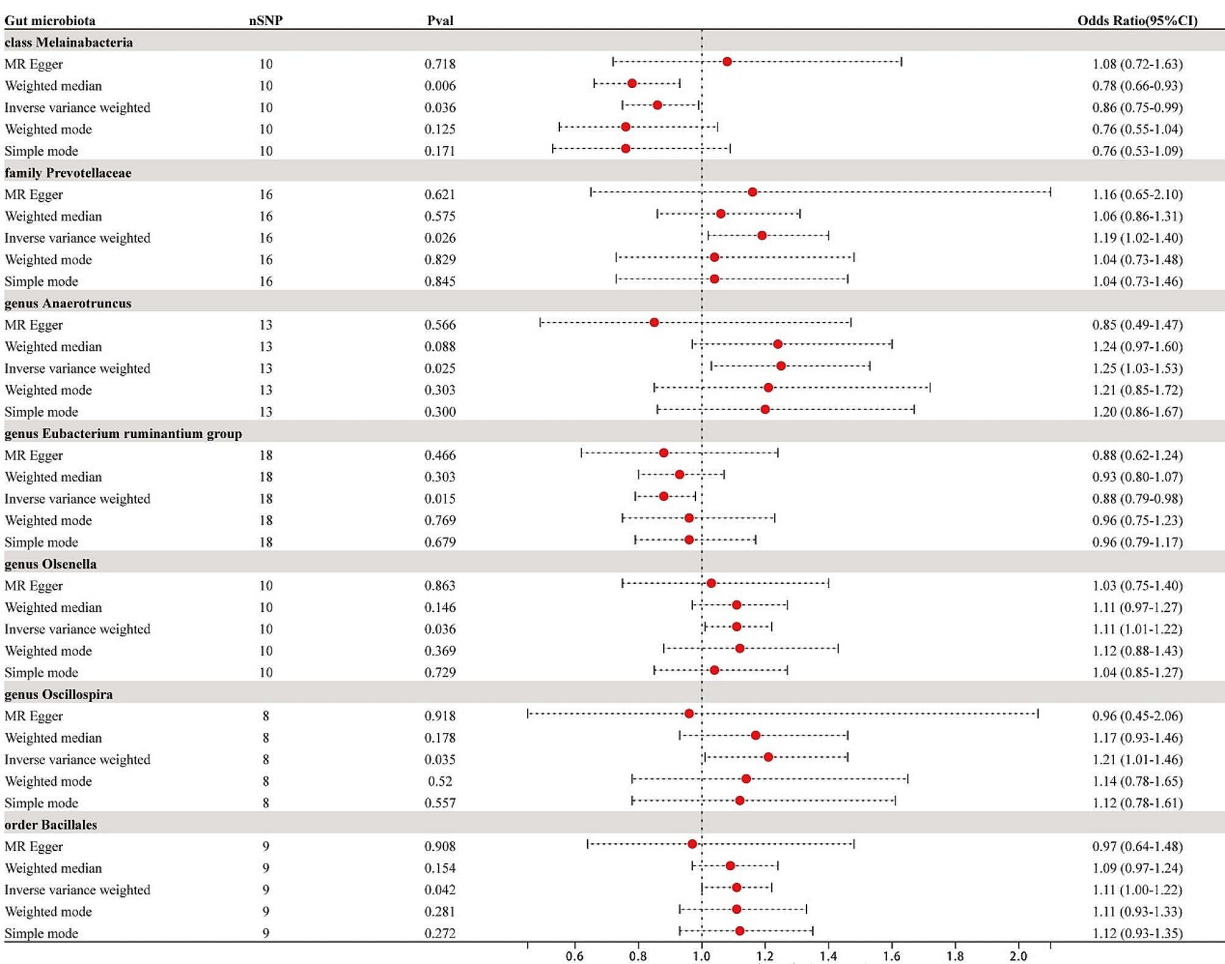
Forrest plot for summary causal effects of gut microbiota on EMs risk based on IVW method for the primary analysis

In this study, 211 gut microbiota relative abundances were selected as the exposure variable from gut microbiota GWAS data involving 18,340 participants. These 211 taxa include 9 phylums, 16 classes, 20 orders, 35 families, and 131 genuses. As both heterogeneity and pleiotropy tests yielded negative results, the IVW analysis results were considered the primary reference indicator. The MR analysis results indicate that seven different gut microbiota at various taxonomic levels (1 class, 1 order, 1 family, and 4 genuses) may be associated with endometriosis, as shown in Fig. 2. The main MR analysis results for the association between all gut microbiota and the risk of EMs, as well as the results of heterogeneity and pleiotropy tests, can be found in Supplementary File 2.

**Fig. 3:**
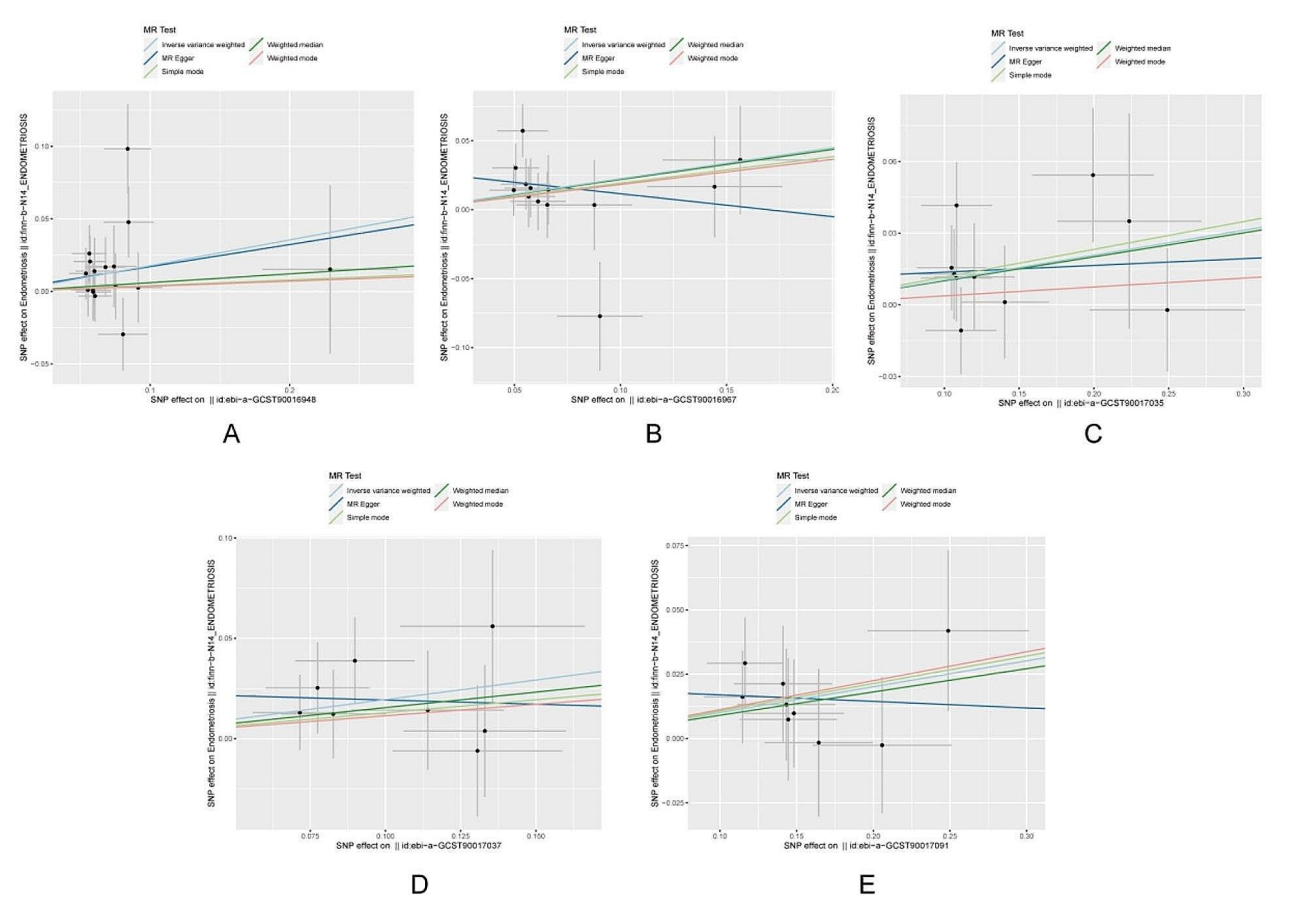
Scatter plots of five taxa of gut microbiota positively associated with EMs. (**A**) family Prevotellaceae (**B**) genus Anaerotruncus (**C**) genus Olsenella (**D**) genus Oscillospira (**E**)order Bacillales

**Fig. 4:**
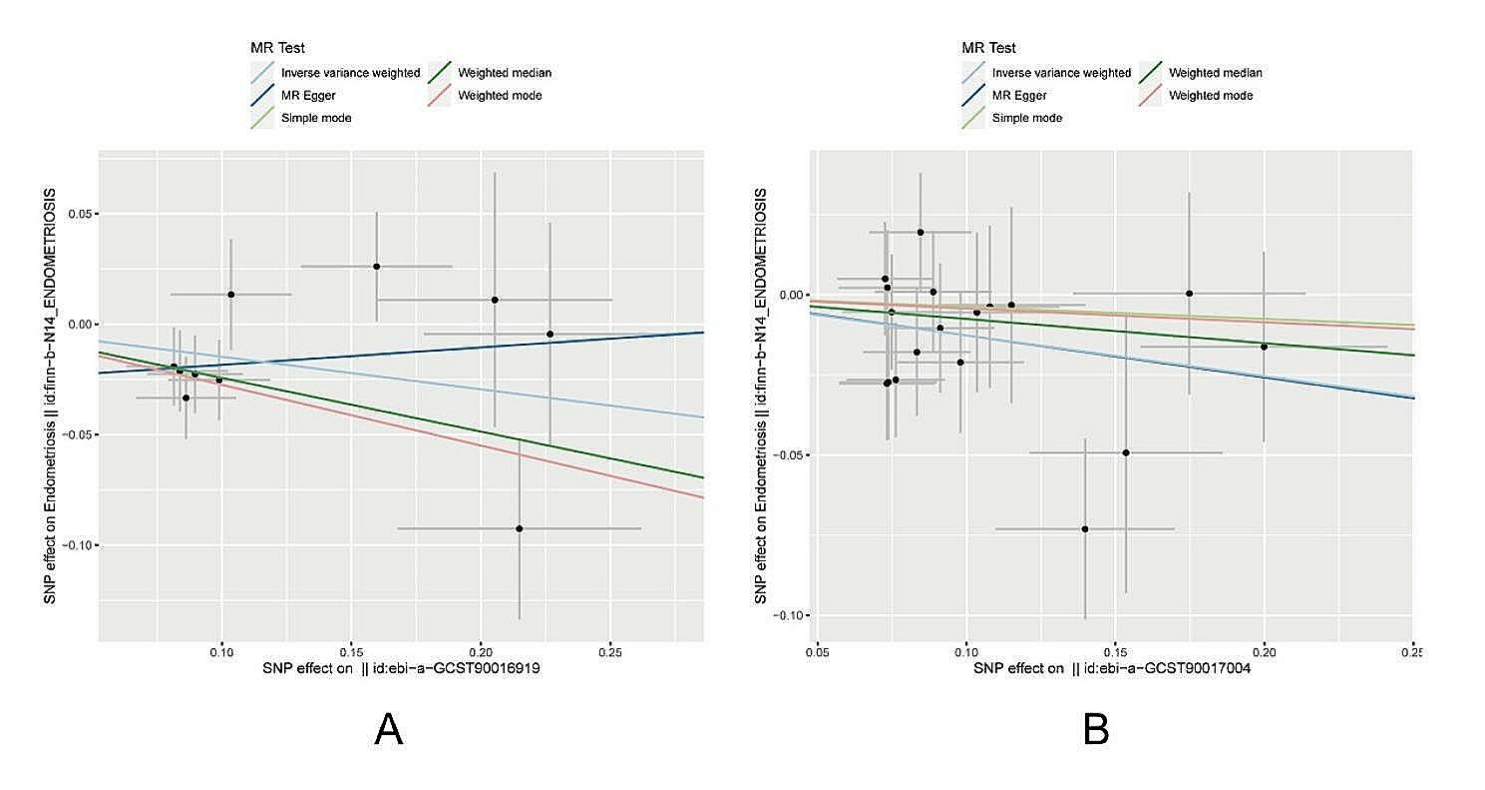
Scatter plots of two taxa of gut microbiota negatively associated with EMs. (**A**) class Melainabacteria (**B**) genus Eubacterium ruminantium group

We identified associations between endometriosis and five microbial taxonomic groups with positive correlations: *family Prevotellaceae* (OR = 1.19, 95%CI 1.02 ∼ 1.40, *P* = 0.026), *genus Anaerotruncus* (OR = 1.25, 95%CI 1.03 ∼ 1.53, *P* = 0.025), *genus Olsenella* (OR = 1.11, 95%CI 1.01 ∼ 1.22, *P* = 0.036), *genus Oscillospira* (OR = 1.21, 95%CI 1.01 ∼ 1.46, *P* = 0.035), *order Bacillales* (OR = 1.11, 95%CI 1.00 ∼ 1.22, *P* = 0.042). Simultaneously, two microbial taxonomic groups showed negative associations with endometriosis: *class Melainabacteria* (OR = 0.86, 95%CI 0.75 ∼ 0.99, *P* = 0.036), *genus Eubacterium ruminantium group* (OR = 0.88, 95%CI 0.79 ∼ 0.98, *P* = 0.015) (Figs. 2, 3 and 4). For detailed results of all SNPs related to these seven gut microbiota (including specific chromosomes, F values, and R^2^), please refer to Supplementary File 3.

**Fig. 5:**
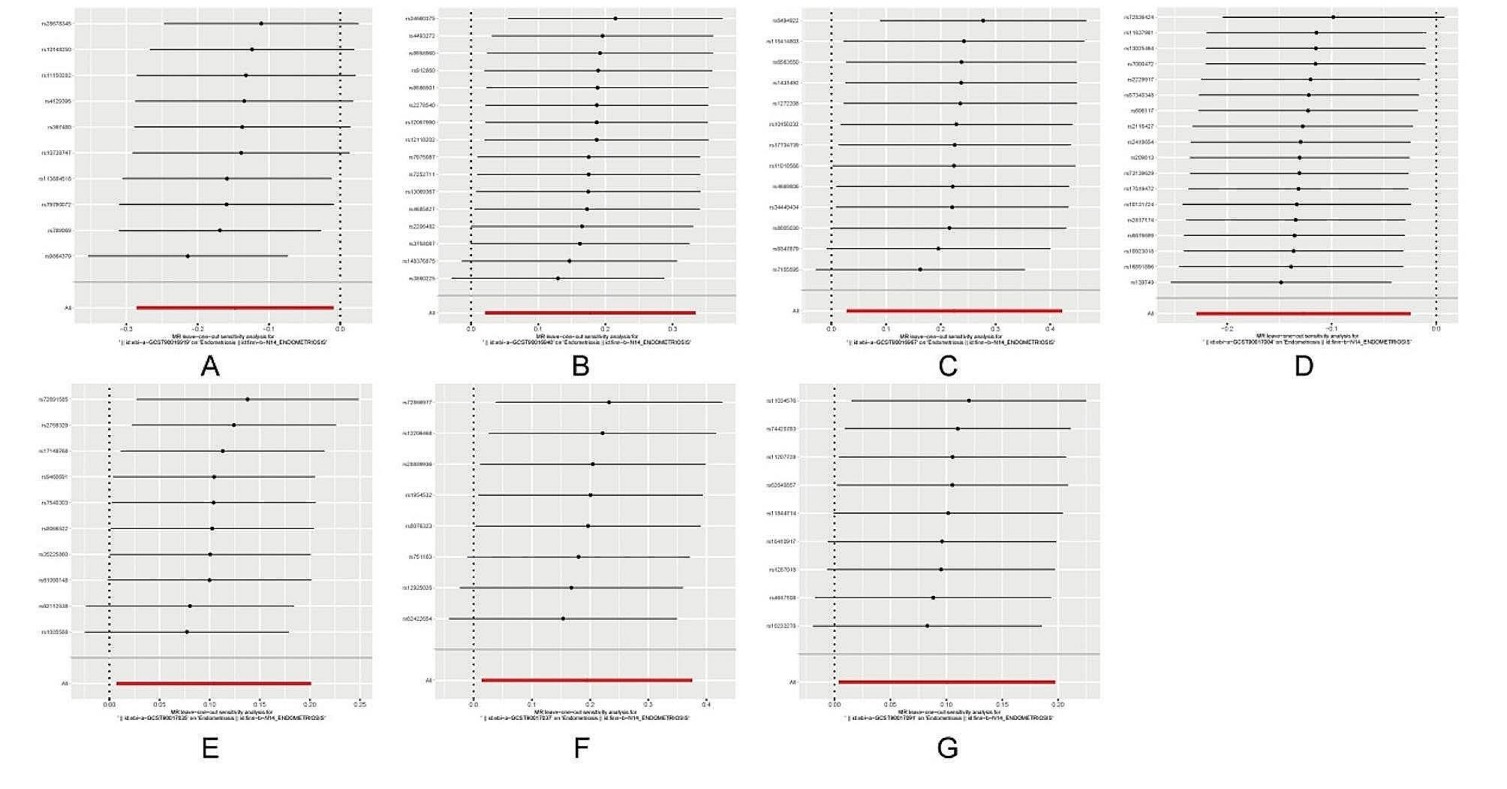
Results of a leave-one-out analysis of the association of gut microbiota with EMs MR. (**A**) class Melainabacteria (**B**) family Prevotellaceae (**C**) genus Anaerotruncus (**D**) genus Eubacterium ruminantium group (**E**) genus Olsenella (**F**) genus Oscillospira (**G**) order Bacillales

As indicated in Supplementary File 3, we noted that the contribution of total variation (R^2^ values) for the 7 gut microbiota ranged from 0.13 to 0.21%, with F values spanning from 18.27 to 29.81. This range effectively rules out the possibility of weak genetic instrumental variables. Heterogeneity testing was conducted with a distribution = 10,000 setting. The Cochran’s Q test for both IVW and MR-Egger regressions indicated the absence of heterogeneity among the SNPs of each microbial taxonomic group. Multiple-effect tests revealed that the MR-Egger regression intercepts were all less than 0.05, and their *P*-values were greater than 0.05, suggesting the absence of horizontal pleiotropy. Furthermore, all MR Steiger directional tests consistently indicated that the direction from gut microbiota to endometriosis was robust for all outcomes (Table 1). Sensitivity analysis was performed using a “leave-one-out” test, and a forest plot was generated. The results indicated that removing any single SNP did not significantly influence the remaining SNP results, all remained on the same side of the null line. This suggests that the MR results in this study are robust. Refer to Fig. 5 for visualization of the sensitivity analysis results.

**Table 1:** Heterogeneity and pleiotropy evaluations for genetically causal associations of gut microbiota with EMs risk

Gut microbiota	nSNP	Cochran’s Q *P*val		MR-Egger		MR Steiger	
		IVW	MR-Egger	egger_intercept	*P*val	Direction	*P*val
class Melainabacteria	10	10.645	0.329	−0.026	0.288	TRUE	1.17E−61
family Prevotellaceae	16	15.496	0.346	0.002	0.933	TRUE	6.98E−56
genus Anaerotruncus	13	13.755	0.405	0.028	0.166	TRUE	6.22E−42
genus Eubacterium ruminantium group	18	12.733	0.692	< 0.001	0.983	TRUE	5.80E−61
genus Olsenella	10	7.374	0.524	0.011	0.629	TRUE	1.16E−33
genus Oscillospira	8	3.269	0.824	0.023	0.555	TRUE	1.22E−27
order Bacillales	9	2.759	0.935	0.020	0.561	TRUE	3.45E−31

### Reverse-direction MR analyses

Finally, a reverse mendelian randomization analysis was conducted, with endometriosis as the exposure factor and gut microbiota as the outcome variables. The results of each SNP of endometriosis and 7 gut microbiota are shown in Supplementary File 4. Heterogeneity and multiple-effect tests yielded negative results. The IVW analysis revealed that there is no causal relationship between endometriosis and the seven different gut microbiota at various taxonomic levels. The MR Steiger directional tests for the 7 gut microbiota with respect to endometriosis yielded TRUE results. Detailed results can be found in Table 2.

**Table 2:** Results of reverse MR analysis of EMs on gut microbiota

Gut microbiota	OR	95%CI	*P*val	Cochran’s Q *P*val	Egger_*P*val	MR Steiger	
						Direction	*P*val
class Melainabacteria	1.012866671	0.927–1.106	0.776483371	0.850	0.305	TRUE	3.15E-14
family Prevotellaceae	1.038144984	0.982–1.098	0.18802718	0.452	0.596	TRUE	3.31E-11
genus Anaerotruncus	0.968896866	0.912–1.030	0.307702166	0.186	0.035	TRUE	3.29E-11
genus Eubacterium ruminantium group	1.041735583	0.962–1.128	0.312730065	0.398	0.620	TRUE	1.38E-11
genus Olsenella	1.101249839	0.987–1.229	0.084877138	0.564	0.766	TRUE	8.30E-12
genus Oscillospira	1.037769604	0.970–1.110	0.279033778	0.474	0.644	TRUE	3.48E-12
order Bacillales	0.998659244	0.999−0.886	0.982427034	0.585	0.586	TRUE	2.52E-12

## Discussion

### Main findings and interpretation

In this study, we assessed for the first time the potential relationship between gut microbiota and endometriosis by a bidirectional MR method, and identified the presence of specific microbial groups at the level of phylum, order, family, and genus that are closely related to EMs, *family Prevotellaceae*, *genus Anaerotruncus*, *genus Olsenella*, *genus Oscillospira* and *order Bacillales* had a risk effect on endometriosis, and *class Melainabacteria*, *genus Eubacterium ruminantium group* was a protective factor against endometriosis. Sensitivity analyses showed no horizontal pleiotropy, indicating that our MR analyses were not affected by confounding factors, and “leave-one-out” analyses confirmed the robustness of the study.

During menstruation, when endometrial tissue retrogrades into the peritoneal cavity and implants into surrounding tissues, such as the intestines or peritoneum, it leads to the formation of endometriotic lesions [26]. In approximately 10% of women, the immune system fails to clear these ectopic endometrial cells, leading to the activation of macrophages, secretion of pro-inflammatory cytokines and growth factors, and the spread of the lesions [27, 28]. The gut microbiota is a crucial component of the human immune system, with immunomodulatory functions mediated through interactions with stromal cells and epithelial cells. Research has shown that microbial metabolites act as messengers between the gut microbiota and immune functions [29–31]. In studies involving mice with endometriosis, alterations in microbial metabolites were observed. The consumption of gut microbiota suppressed inflammation related to endometriosis [32] and influenced immune cell populations, suggesting that gut microbiota can influence endometriosis through immune pathways.

The abnormal endocrine microenvironment within EMs lesions is considered a key characteristic of endometriosis. Estrogen [33] has a direct cell anti-apoptotic and proliferative effect on EMs lesions and promotes the formation of a pro-inflammatory microenvironment, contributing to the chronic progression of the disease. Estrogen is a major regulatory factor for gut microbiota, and the gut microbiome’s genetic repertoire involved in estrogen metabolism is often referred to as the “estrobolome” [34]. It participates in estrogen regulation by secreting beta-glucuronidase [35], forming the estrogen-gut microbiota axis. Research has shown significant differences in the expression of 17β-estradiol, 16-keto-17β-estradiol, 2-hydroxyestrone, and 2-hydroxyestradiol in individuals with EMs. Additionally, there is a clear positive correlation between the gut microbiota of EMs patients and urinary estrogen levels [36]. *Family Prevotellaceae* belongs to the *Bacteroidetes phylum*, and a meta-analysis [37] found that the abundance of *Bacteroidetes* is positively correlated with estrogen levels. When the *Firmicutes/Bacteroidetes* ratio in the gut decreases, there is an increase in the secretion of beta-glucuronidase in the intestine, leading to elevated estrogen levels. High estrogen levels are directly associated with the development of EMs, and our study provides similar findings.

Multiple studies have indicated [7, 33] that individuals with endometriosis experience dysbiosis in their gut microbiota. The gut microbiota, when fermenting carbohydrates, produces short-chain fatty acids (SCFAs) that can activate G protein-coupled receptors. This activation has beneficial effects by reducing food intake, improving insulin sensitivity, inhibiting fat accumulation, and reducing systemic inflammation [38]. However, in cases of gut microbiota dysbiosis, there is a reduction in SCFA production. Simultaneously, certain neuroactive metabolites, such as glutamate and butyric acid, increase in level. These metabolites can stimulate brain neurons and, through the hypothalamus-pituitary-ovary axis, increase ovarian estrogen secretion, exacerbating the condition of patients [39, 40].

It is noteworthy that PERROTTA et al. [41] established an EM classification model based on random forest, revealing that the vaginal microbiota could predict the severity of endometriomas (EMs), with *Anaerococcus* identified as the most crucial factor, while the gut microbiota lacked corresponding accuracy. Furthermore, CHEN et al. [42] built a model based on the female reproductive tract microflora, which can distinguish whether infertility is caused by EMs. Considering the potential influences on the gut microbiota from factors such as diet, antimicrobial drugs, and psychological stress, relying on it as a tool for early diagnosis and screening of EMs is unreliable. Similarly, the reproductive tract microbiota can be affected by different physiological stages and diseases like vaginal infections. Therefore, exploration of non-invasive diagnostic methods for EMs is still needed, and using saliva for diagnosis may be more helpful [43]. However, what can be confirmed is the causal association between gut microbiota and endometriosis, with a dynamic interplay between the two, which holds potential implications for future bacteria-based therapies.

### Limitation

However, our study has several limitations: (1) Human behavior is complex, and while understanding the genetic risk of a disease can help prevent its occurrence to some extent, environmental factors also play a role in the development of the disease [44], and MR can only partially eliminate the interference of confounding factors such as the environment [45]. (2) The current study may not comprehensively explore the entire spectrum of the gut microbiota, from phylum to genus level, potentially missing other microbial taxa that could have a causal relationship with endometriosis, especially those associated with increased risk. (3) The outcome data used in the study is derived from European populations, and caution should be exercised when extrapolating the results to other populations with different lifestyles, cultural backgrounds, and genetic backgrounds, as specific traits may vary across different racial and ethnic groups driven by their distinct living environments and genetic backgrounds. Efforts should be made to include populations of all ethnicities globally in genetic studies of this nature. (4) Although we have demonstrated a causal relationship between gut microbiota and endometriosis, the underlying mechanism is still unclear and requires further research.

## Conclusions

The study collected data from GWAS databases and used a two-sample bidirectional MR approach to confirm the potential causal relationship between gut microbiota and endometriosis, providing new insights into the pathogenesis and treatment of endometriosis. Future research should aim to further elucidate the underlying mechanisms by which these microbial communities influence endometriosis, explore potential treatment strategies targeting gut microbiota.

### Electronic supplementary material

Below is the link to the electronic supplementary material.

Supplementary Material 1

Supplementary Material 2

Supplementary Material 3

Supplementary Material 4

## Data Availability

All data generated or analysed during this study are included in this published article and its supplementary information files.

## Abbreviations

MR: Mendelian randomization
EMs: Endometriosis
RCT: Randomized controlled trials
SNPs: Single nucleotide polymorphisms
IVs: Instrumental variables
GWAS: Genome wide association study
LD: Linkage disequilibrium
IVW: Inverse variance weighted
WM: Weighted median
