# Supplementary material for: Assessing the relationship between gut microbiota and endometriosis: a bidirectional two-sample mendelian randomization analysis
Source: BMC Womens Health. 2024 Feb 16;24:123. doi: 10.1186/s12905-024-02945-z (PMC10873948; doi:10.1186/s12905-024-02945-z)
Supplement: Supplementary file 1 — Supplementary Material 1 [file 12905_2024_2945_MOESM1_ESM.pdf]

Supplementary File 1. Participant descriptions per cohort in dataset of gut microbiota

** Shannon Diversity Index

Study name	Study design	Ethnicity	Country	Nsamples GWAS	Trait	Study Population						Males						Females					
						n	mean	SD	median	min	max	n	mean	SD	median	min	max	n	mean	SD	median	min	max
	Populatior	European	Germany	721	Age (yrs)	721	61.53	12.62	63.20	25.10	82.50	396	62.13	11.90	63.00	25.20	82.50	325	60.79	13.44	63.80	25.10	82.00
**BSPSPC**					BMI (kg/m2)	721	27.32	4.52	26.77	17.77	49.54	396	27.62	3.89	27.08	17.40	45.51	325	26.90	5.17	26.25	17.77	49.53
					alpha-diversity**	721	2.99	0.36	3.03	1.12	3.84	396	2.99	0.37	3.05	1.12	3.65	325	2.98	0.35	3.02	1.57	3.84
	Populatior	European	Germany	721	Age (yrs)	266	55.79	3.15	56.00	48.00	61.00	127	55.64	3.00	56.00	48.00	60.00	139	55.92	3.29	57.00	48.00	61.00
**Cardia Microbiome-W**					BMI (kg/m2)	266	27.74	5.35	26.72	17.79	49.26	127	28.89	4.59	27.83	20.90	49.26	139	26.70	5.78	25.32	17.79	47.11
					alpha-diversity**	266	2.72	0.44	2.77	1.50	3.72	127	2.67	0.48	2.69	1.50	3.72	139	2.76	0.41	2.81	1.68	3.53
	Children	European	Denmark	380	Age (yrs)	380	4.37	0.78	4.00	4.00	6.00	201	4.44	0.83	4.00	4.00	6.00	179	4.30	0.72	4.00	4.00	6.00
**COPSAC**					BMI (kg/m2)	371	15.59	1.05	15.58	12.77	20.24	196	15.59	0.93	15.58	13.23	18.50	175	15.58	1.17	15.60	12.77	20.24
					alpha-diversity**	380	2.62	0.45	2.68	0.89	3.43	201	2.61	0.49	2.69	0.89	3.43	179	2.64	0.41	2.67	1.18	3.37
	Children	European	Denmark	380	Age (yrs)	2396	54.90	11.51	56.00	18.00	72.00	1127	56.00	11.41	58.00	18.00	72.00	1269	53.90	11.50	55.00	19.00	72.00
**DanFunD**					BMI (kg/m2)	2396	26.10	4.32	25.50	16.80	49.40	1127	26.70	3.80	26.20	18.20	49.40	1269	25.50	4.67	24.50	16.80	47.60
					alpha-diversity**	2396	3.48	0.28	3.52	2.16	4.10	1127	3.48	0.28	3.52	2.37	4.09	1269	3.47	0.28	3.52	2.16	4.08
	Populatior	European	Belgian	2259	Age	2259	50.85	14.31	54.00	16.00	88.00	911	53.58	13.82	57.00	16.00	85.00	1348	49.01	13.82	51.00	16.00	88.00
**FGFP**					BMI	2259	24.97	4.43	24.34	15.06	55.83	911	25.91	4.16	25.53	15.26	43.71	1348	24.35	4.50	23.47	15.06	55.83
					alpha-diversity**	2259	2.56	0.32	2.58	0.60	3.40	911	2.55	0.31	2.56	1.47	3.35	1348	2.56	0.33	2.59	0.60	3.40
	Populatior	European	Germany	960	Age (yrs)	960	51.40	14.60	52.00	16.00	81.00	404	53.70	13.80	54.00	18.00	80.00	556	49.80	15.00	50.00	16.00	81.00
**FOCUS**					BMI (kg/m2)	960	26.40	5.26	25.60	14.30	60.20	404	27.20	4.54	26.70	16.10	54.60	556	25.80	5.65	24.60	14.30	60.20
					alpha-diversity**	960	2.65	0.44	2.69	0.57	3.64	404	2.66	0.44	2.70	1.12	3.64	556	2.64	0.43	2.69	0.57	3.53
	Populatior	European	Canada	378	Age (yrs)	378	18.61	7.79	18.00	6.00	35.00	171	17.33	7.91	16.00	6.00	35.00	207	19.67	7.55	20.00	6.00	35.00
**GEM_HCE_v12**					BMI (kg/m2)	343	22.32	5.13	21.48	13.00	46.26	150	22.21	5.16	21.49	13.00	39.74	193	22.40	5.11	21.48	13.00	46.26
					alpha-diversity**	378	2.97	0.37	3.03	1.55	3.71	171	2.99	0.35	3.03	2.00	3.67	207	2.95	0.38	3.02	1.55	3.71
	Populatior	European	Canada	662	Age (yrs)	662	20.85	7.94	21.00	6.00	35.00	297	19.60	7.95	18.00	6.00	35.00	365	21.87	7.80	22.00	6.00	35.00
**GEM_ICHIP_ HCE**					BMI (kg/m2)	647	23.37	5.82	22.39	12.77	55.00	288	23.36	5.61	22.56	13.32	55.00	359	23.38	6.00	22.21	12.77	53.26
					alpha-diversity**	662	2.97	0.39	3.03	1.48	3.81	297	2.95	0.38	3.00	1.48	3.81	365	2.98	0.39	3.04	1.60	3.67
	Populatior	European	The Nethe	875	Age (yrs)	875	45.09	13.30	45.42	18.00	81.00	371	45.28	13.40	45.00	18.00	81.00	504	44.95	13.23	45.42	18.00	80.00
**LLD**					BMI (kg/m2)	875	25.24	4.16	24.59	16.67	48.56	371	25.54	3.53	25.25	16.67	48.56	504	25.01	4.55	23.94	16.88	42.25
					alpha-diversity**	875	3.22	0.29	3.25	2.00	3.90	371	3.20	0.29	3.24	2.00	3.83	504	3.24	0.28	3.25	2.02	3.90
	Populatior	European	Finland	522	Age (yrs)	522	61.91	5.42	62.70	50.40	70.20	522	61.91	5.42	62.70	50.40	70.20	0	0.00	0.00	0.00	0.00	0.00
**METSIM**					BMI (kg/m2)	522	27.92	3.61	27.36	19.12	39.32	522	27.92	3.61	27.36	19.12	39.32	0	0.00	0.00	0.00	0.00	0.00
					alpha-diversity**	522	2.83	0.44	2.89	1.03	3.67	522	2.83	0.44	2.89	1.03	3.67	0	0.00	0.00	0.00	0.00	0.00
	Populatior	European	The Nethe	80	Age (yrs)	80	48.74	18.15	56.00	19.00	71.00	34	49.94	17.38	53.00	21.00	69.00	46	47.85	18.83	57.50	19.00	71.00
**MIBS**					BMI (kg/m2)	75	24.25	3.66	23.50	17.30	38.70	32	25.17	3.12	24.90	19.80	32.50	43	23.56	3.92	22.90	17.30	38.70
					alpha-diversity**	80	3.27	0.36	3.31	2.05	3.95	34	3.33	0.36	3.35	2.58	3.95	46	3.23	0.36	3.24	2.05	3.80
	Populatior	European	USA	77	Age (yrs)	77	71.90	7.52	72.00	55.00	88.00	32	73.38	6.72	74.00	62.00	87.00	45	70.84	7.94	70.00	55.00	88.00
**NGRC**					BMI (kg/m2)	74	27.83	5.43	26.44	18.02	43.04	32	28.65	5.44	26.56	22.47	43.04	42	27.21	5.41	25.78	18.02	40.93
					alpha-diversity**	77	2.37	0.52	2.36	1.22	3.34	32	2.40	0.55	2.42	1.22	3.32	45	2.36	0.51	2.34	1.46	3.34
	MZ Twins !	European	The Nethe	279	Age (yrs)	279	35.38	11.68	35.00	19.00	68.00	83	37.38	12.60	37.00	19.00	61.00	196	34.54	11.19	34.00	19.00	68.00
**NTR**					BMI (kg/m2)	277	25.02	4.67	25.10	17.38	43.89	83	25.90	3.83	25.55	19.09	43.10	194	24.65	4.95	23.24	17.38	43.89
					alpha-diversity**	279	3.31	0.34	3.36	1.92	4.03	83	3.29	0.40	3.33	1.92	4.03	196	3.32	0.31	3.38	1.99	3.93
	Populatior	European	Sweden	134	Age (yrs)	134	54.75	11.26	57.00	22.00	71.00	51	54.78	9.97	56.00	31.00	71.00	83	54.72	12.05	57.00	22.00	71.00
**POPCOL**					BMI (kg/m2)	134	24.50	3.32	24.07	16.90	34.82	51	25.48	3.37	24.76	20.15	34.82	83	23.91	3.17	23.51	16.90	33.33
					alpha-diversity**	134	3.04	0.24	3.07	2.23	3.44	51	3.03	0.27	3.07	2.23	3.43	83	3.05	0.22	3.08	2.37	3.44
	Populatior	European	The Nethe	1220	Age (yrs)	1220	62.30	5.87	57.00	45.60	87.50	515	62.20	5.78	56.90	45.60	87.10	705	62.37	5.93	57.10	45.80	87.50
**RS3**					BMI (kg/m2)	1220	27.43	4.51	26.80	15.80	53.30	515	27.62	3.95	26.90	16.80	42.60	705	27.34	4.88	26.70	15.80	53.30
					alpha-diversity**	1220	3.15	0.39	3.21	0.77	3.86	515	3.15	0.36	3.20	1.75	3.86	705	3.15	0.41	3.21	0.77	3.86
	Populatior	European	Germany	996	Age (yrs)	996	56.93	13.52	57.00	31.00	89.00	462	57.75	13.51	59.00	31.00	89.00	534	56.22	13.50	56.50	31.00	88.00
**SHIP**					BMI (kg/m2)	996	28.06	4.68	27.51	17.25	48.64	462	28.49	3.92	27.99	18.82	48.64	534	27.68	5.22	26.60	17.25	43.49
					alpha-diversity**	996	2.91	0.41	2.96	0.39	3.75	462	2.89	0.41	2.95	1.42	3.66	534	2.93	0.40	2.97	0.39	3.75
	Populatior	European	Germany	905	Age (yrs)	905	50.24	13.69	51.00	20.00	81.00	396	50.21	14.31	50.00	22.00	80.00	509	50.26	13.20	51.00	20.00	81.00
**SHIP-TREND**					BMI (kg/m2)	905	27.38	4.55	27.07	17.74	53.63	396	27.84	3.80	27.76	17.74	43.88	509	27.02	5.02	26.56	18.49	53.63
					alpha-diversity**	905	2.90	0.39	2.96	1.19	3.80	396	2.93	0.36	2.96	1.19	3.78	509	2.88	0.40	2.96	1.27	3.80
	Twins Stuc	European	UK	1205	Age (yrs)	1205	61.52	10.72	63.00	24.00	89.00	104	62.39	10.67	62.00	35.00	84.00	1101	61.44	10.73	63.00	24.00	89.00
**TwinsUK**					BMI (kg/m2)	1205	26.26	4.94	25.36	16.23	50.29	104	27.07	4.09	26.80	19.76	44.39	1101	26.18	5.01	25.25	16.23	50.29
					alpha-diversity**	1205	2.74	0.50	2.85	0.64	3.67	104	2.74	0.47	2.83	1.28	3.43	1101	2.74	0.51	2.86	0.64	3.66
