# Supplementary material for: Assessing the relationship between gut microbiota and endometriosis: a bidirectional two-sample mendelian randomization analysis
Source: BMC Womens Health. 2024 Feb 16;24:123. doi: 10.1186/s12905-024-02945-z (PMC10873948; doi:10.1186/s12905-024-02945-z)
Supplement: Supplementary file 2 — Supplementary Material 2 [file 12905_2024_2945_MOESM2_ESM.pdf]

Supplementary File 2. MR analysis results for all gut microbiota and EMs

	id.exposure	nsnp	b_MR.Egger	se_MR.Egger	pval_MR.Egger	b_WME	se_WME	pval_WME	b_IVW	se_IVW	pval_IVW	Q_MR.Egger	Q_pval_MR.Egger	Q_IVW	Q_pval_IVW	egger_intercept	se	pval
**1**	ebi-a-GCST	15	-0.37035889	0.25465991	0.16957118	-0.0089746	0.11930156	0.94003509	-0.0038	0.09214	0.966688	16.8839833	0.204662126	19.9388	0.13208131	0.027735781	0.018	0.149083
**2**	ebi-a-GCST	7	0.23837349	0.34951551	0.525536901	0.0975282	0.11575315	0.39947831	0.07895	0.09319	0.396867	1.16784261	0.947924977	1.39181	0.96635444	-0.015012432	0.032	0.65597
**3**	ebi-a-GCST	18	-0.0000115	0.21628825	0.999958253	0.04065711	0.10695746	0.70385328	0.03697	0.07865	0.638311	7.86937105	0.952660344	7.90307	0.96857561	0.002918436	0.016	0.856664
**4**	ebi-a-GCST	14	0.006872115	0.17934395	0.970064178	-0.0470065	0.12000442	0.69527536	0.01736	0.08811	0.843808	7.09692517	0.851142373	7.10143	0.8968489	0.000950705	0.014	0.947581
**5**	ebi-a-GCST	11	-0.2805235	0.33856524	0.428775508	0.05658843	0.13334647	0.67129497	0.14089	0.10134	0.164457	7.88786197	0.54548544	9.58963	0.47720269	0.029973735	0.023	0.224427
**6**	ebi-a-GCST	12	0.069642804	0.2630109	0.796548578	-0.0840593	0.12478734	0.50055264	-0.0355	0.09389	0.705365	10.7216629	0.379610222	10.9201	0.44998017	-0.007928609	0.018	0.676158
**7**	ebi-a-GCST	13	0.184344395	0.56779246	0.751522562	-0.149476	0.14918548	0.31636904	-0.0094	0.12864	0.941593	20.9415474	0.033981723	21.1763	0.04785812	-0.014299089	0.041	0.732125
**8**	ebi-a-GCST	13	0.140508381	0.26213523	0.60261836	0.06310283	0.13151742	0.63136547	0.00756	0.09611	0.937303	14.8088279	0.191417243	15.2128	0.23000568	-0.010436028	0.019	0.594786
**9**	ebi-a-GCST	13	-0.47733768	0.43787345	0.298961548	-0.0680927	0.14065073	0.62829612	-0.1489	0.101	0.140427	6.89088111	0.80785005	7.48513	0.82396196	0.020479559	0.027	0.457019
**10**	ebi-a-GCST	6	0.075143894	0.53356276	0.894808536	0.17614833	0.17197142	0.30569907	0.09363	0.15807	0.553638	6.52790051	0.163040893	6.5301	0.25799682	0.001436893	0.039	0.97247
**11**	ebi-a-GCST	8	-0.29128274	0.22878541	0.250059394	-0.0142538	0.08378565	0.8649137	-0.0374	0.06436	0.561665	4.36436164	0.627495617	5.70212	0.57492895	0.037657022	0.033	0.291395
**12**	ebi-a-GCST	10	0.078651113	0.2103535	0.718193911	-0.2431089	0.08897973	0.00629161	-0.1475	0.0705	0.036476	9.16094468	0.328901421	10.6455	0.30079454	-0.026259488	0.023	0.287818
**13**	ebi-a-GCST	10	-0.00194738	0.21780983	0.993085343	0.03586008	0.06880263	0.60222595	0.00572	0.05292	0.913884	5.70097812	0.680685972	5.7023	0.76930427	0.00127566	0.035	0.971931
**14**	ebi-a-GCST	12	-0.42869172	0.27329965	0.147817763	-0.0801239	0.11421811	0.48299175	-0.0888	0.08207	0.279502	9.87193689	0.451799736	11.5642	0.39727496	0.030986029	0.024	0.222479
**15**	ebi-a-GCST	12	-0.02519169	0.5585548	0.964914299	0.23210036	0.15535045	0.1351642	0.09391	0.15469	0.543801	25.795766	0.0040248	25.9238	0.00666146	0.007863873	0.035	0.828184
**16**	ebi-a-GCST	11	-0.23628501	0.28369711	0.426462538	0.06110554	0.10948561	0.57676598	0.10912	0.08416	0.194793	3.12339598	0.959200939	4.7488	0.90731443	0.028395139	0.022	0.234268
**17**	ebi-a-GCST	7	-0.34558008	0.29551507	0.294945073	-0.0029589	0.12182341	0.98062229	0.02962	0.09759	0.76152	3.17151272	0.673562944	4.98084	0.54627263	0.039440152	0.029	0.236373
**18**	ebi-a-GCST	5	0.043295418	0.29138885	0.891308221	-0.029209	0.12055208	0.80855278	0.03694	0.11091	0.739067	5.21544494	0.156684103	5.21647	0.26579874	-0.000758338	0.031	0.982161
**19**	ebi-a-GCST	11	0.229332667	0.59619554	0.709420997	-0.1818651	0.15304222	0.23470235	-0.1466	0.12601	0.244594	13.5898693	0.137680532	14.2202	0.16318246	-0.024470065	0.038	0.534342
**20**	ebi-a-GCST	9	0.588512203	0.71518777	0.437707712	0.00570812	0.15725078	0.97104358	-0.1204	0.13804	0.382936	10.5587575	0.159057353	12.0982	0.14687663	-0.046863876	0.046	0.346021
**21**	ebi-a-GCST	8	0.066499303	0.51653155	0.901768858	0.08799763	0.11994107	0.46314751	0.07684	0.11882	0.517833	15.9542385	0.014001707	15.9554	0.02552721	0.001047655	0.051	0.984177
**22**	ebi-a-GCST	12	-0.57671968	0.28615451	0.071525753	-0.0211065	0.1282454	0.86927519	-0.0504	0.09716	0.603658	11.3899298	0.327956358	15.6347	0.15524487	0.040741954	0.021	0.082369
**23**	ebi-a-GCST	12	-0.57671968	0.28615451	0.071525753	-0.0211065	0.1282454	0.86927519	-0.0504	0.09716	0.603658	11.3899298	0.327956358	15.6347	0.15524487	0.040741954	0.021	0.082369
**24**	ebi-a-GCST	10	0.054932639	0.29192184	0.855424425	-0.0183355	0.13413432	0.89127168	0.08732	0.09654	0.365744	8.580504	0.378909191	8.59546	0.47542217	0.00252555	0.021	0.908905
**25**	ebi-a-GCST	15	0.003032463	0.18489829	0.987163753	-0.0918587	0.09210208	0.31859108	-0.0853	0.06753	0.206671	12.0683116	0.522050415	12.3315	0.57969613	-0.008636546	0.017	0.616516
**26**	ebi-a-GCST	13	0.184344395	0.56779246	0.751522562	-0.149476	0.14791529	0.31223123	-0.0094	0.12864	0.941593	20.9415474	0.033981723	21.1763	0.04785812	-0.014299089	0.041	0.732125
**27**	ebi-a-GCST	11	-0.07115227	0.25549505	0.786930531	-0.104981	0.09551683	0.2717317	-0.1142	0.07468	0.126108	4.15636785	0.900822444	4.18746	0.93849425	-0.004589289	0.026	0.863937
**28**	ebi-a-GCST	10	0.09296954	0.28878297	0.755752693	0.06224067	0.14251106	0.66229792	0.04222	0.11274	0.708025	13.2475498	0.103611295	13.3092	0.14910685	-0.004316205	0.022	0.851799
**29**	ebi-a-GCST	7	0.133034447	0.72201874	0.861054455	-0.019872	0.14722861	0.89263237	-0.0059	0.12091	0.961151	2.83833552	0.724892732	2.87643	0.82418932	-0.010264049	0.053	0.852946
**30**	ebi-a-GCST	13	-0.47733768	0.43787345	0.298961548	-0.0680927	0.132934	0.60849027	-0.1489	0.101	0.140427	6.89088111	0.80785005	7.48513	0.82396196	0.020479559	0.027	0.457019
**31**	ebi-a-GCST	8	-0.64486162	0.32926408	0.097899375	-0.0384377	0.06906462	0.57783661	-0.0146	0.05794	0.80138	5.03924018	0.53879022	8.79608	0.26763023	0.084380852	0.044	0.100685
**32**	ebi-a-GCST	9	0.695046225	0.44512142	0.162381221	0.16911947	0.15691055	0.28111938	0.17528	0.11646	0.132304	5.21137715	0.634185242	6.67508	0.57206016	-0.034569367	0.029	0.265609
**33**	ebi-a-GCST	16	-0.07677445	0.32094081	0.814403985	0.01772016	0.12673905	0.88880533	-0.049	0.09213	0.595062	10.5927286	0.717692325	10.6009	0.78033824	0.00173864	0.019	0.929212
**34**	ebi-a-GCST	8	0.293044004	0.22312723	0.23705172	0.10579605	0.09497029	0.26528303	0.08988	0.08907	0.312915	9.71400416	0.137224147	11.3113	0.12560673	-0.027935992	0.028	0.358936
**35**	ebi-a-GCST	10	-0.00194738	0.21780983	0.993085343	0.03586008	0.070637	0.61168688	0.00572	0.05292	0.913884	5.70097812	0.680685972	5.7023	0.76930427	0.00127566	0.035	0.971931
**36**	ebi-a-GCST	14	0.032699503	0.21821174	0.883370427	-0.1182349	0.06947601	0.08879129	-0.0754	0.05481	0.168787	15.9734635	0.192454506	16.3237	0.23209304	-0.015105906	0.029	0.617319
**37**	ebi-a-GCST	14	0.081207227	0.13209594	0.550193077	0.01683061	0.08645187	0.84564205	0.00221	0.06073	0.970925	11.832524	0.459221975	12.286	0.50434595	-0.010457521	0.016	0.513467
**38**	ebi-a-GCST	9	0.294792375	0.23353639	0.247268734	0.00962175	0.11133843	0.9311334	-0.0416	0.08401	0.620764	5.42105361	0.608722586	7.80378	0.45286882	-0.032567696	0.021	0.166594
**39**	ebi-a-GCST	13	0.215819304	0.17981528	0.255261356	0.02026213	0.1102546	0.85418932	0.09371	0.07909	0.236073	8.76682447	0.643405574	9.33858	0.67377099	-0.010757662	0.014	0.465446
**40**	ebi-a-GCST	9	0.59474766	0.77368371	0.467196777	0.22152978	0.18239692	0.2245386	0.24482	0.16601	0.140293	13.5292747	0.06021574	13.9459	0.0831858	-0.02098042	0.045	0.656528
**41**	ebi-a-GCST	16	0.152537721	0.30131611	0.620567442	0.06040231	0.10763028	0.57465991	0.17748	0.07995	0.026431	15.4882081	0.345616288	15.4964	0.41628575	0.001789265	0.021	0.932614
**42**	ebi-a-GCST	15	0.015160501	0.258985	0.954210241	-0.0035209	0.08207159	0.96578083	-0.0467	0.06087	0.443363	10.2433233	0.673926173	10.3036	0.73966471	-0.006298634	0.026	0.809844
**43**	ebi-a-GCST	17	-0.22398054	0.27793809	0.432911267	-0.164583	0.11575121	0.15506444	-0.1203	0.08704	0.167059	17.7078073	0.278338428	17.8912	0.33030741	0.007798535	0.02	0.699034
**44**	ebi-a-GCST	9	-0.41726997	0.29066391	0.194261143	-0.138036	0.14938669	0.35547686	-0.155	0.12908	0.22979	11.3565852	0.123803566	13.0011	0.11181226	0.027861154	0.028	0.347559
**45**	ebi-a-GCST	13	0.40880441	0.55977138	0.480468178	0.10896509	0.13093274	0.40528373	0.12313	0.13517	0.362343	24.3827319	0.011212665	24.9982	0.01483122	-0.022330445	0.042	0.608689
**46**	ebi-a-GCST	9	-0.07032284	0.26325841	0.797071896	-0.0360438	0.10310419	0.72664925	-0.0743	0.08236	0.36713	11.7478464	0.109161987	11.7483	0.16279363	-0.000478021	0.03	0.987721
**47**	ebi-a-GCST	13	-0.23575237	0.25108874	0.367931191	-0.0066396	0.10053129	0.94734155	0.03358	0.08041	0.676248	12.8814523	0.301144231	14.3789	0.2771671	0.0231698	0.02	0.282181
**48**	ebi-a-GCST	13	-0.13807559	0.21928794	0.541777957	0.05775626	0.0723052	0.4244155	0.05718	0.05134	0.265427	9.72879578	0.554934609	10.5676	0.56629338	0.02310492	0.025	0.379381
**49**	ebi-a-GCST	19	-0.0320381	0.13745349	0.818480125	-0.130208	0.09766112	0.18244529	-0.061	0.06773	0.368131	15.5204604	0.558047119	15.5789	0.62190799	-0.002573614	0.011	0.81183
**50**	ebi-a-GCST	11	-0.23570989	0.28367901	0.427520408	0.06107786	0.10766457	0.57051196	0.10919	0.08418	0.194633	3.12668065	0.959059811	4.7476	0.90738846	0.028347149	0.022	0.234864
**51**	ebi-a-GCST	12	-0.17124265	0.28458899	0.560754409	0.05294189	0.06777581	0.43472456	-0.0171	0.05788	0.768028	16.9313708	0.07589643	17.4512	0.09521931	0.022666528	0.041	0.591684
**52**	ebi-a-GCST	7	-0.10371197	0.25551904	0.701611919	-0.024474	0.11075757	0.82511633	0.05669	0.09989	0.570338	8.20140381	0.145479614	8.97786	0.17482714	0.019829411	0.029	0.522053
**53**	ebi-a-GCST	8	0.540053784	0.39258162	0.218077252	0.22249662	0.11528857	0.05361794	0.08729	0.08998	0.332011	6.0246178	0.420438038	7.43002	0.38551891	-0.040995723	0.035	0.281529
**54**	ebi-a-GCST	11	-0.23476592	0.28351393	0.429048431	0.06096222	0.10770802	0.57139704	0.10921	0.08415	0.194373	3.13140163	0.958856468	4.74563	0.90750956	0.028282408	0.022	0.235757
**55**	ebi-a-GCST	13	-0.82402401	0.53502728	0.151780894	0.01270048	0.14085215	0.92815304	-0.0343	0.11655	0.768236	12.257686	0.344588845	14.794	0.25289542	0.047375346	0.031	0.159557
**56**	ebi-a-GCST	8	-0.9028253	0.49434596	0.117589022	-0.1404455	0.07517882	0.06174091	-0.0398	0.0833	0.633041	12.0094197	0.061758992	18.2462	0.01090742	0.123933469	0.07	0.127972
**57**	ebi-a-GCST	6	-0.62028502	0.80995231	0.486471104	-0.0362246	0.08182384	0.65797267	-0.0028	0.08339	0.973567	8.4921834	0.075124515	9.74069	0.08292506	0.086557881	0.113	0.485922
**58**	ebi-a-GCST	11	0.029075096	0.33994926	0.933714276	-0.0096542	0.0808938	0.90500231	-0.0084	0.0594	0.887964	10.4482307	0.315430805	10.4628	0.40086933	-0.004218602	0.038	0.913236
**59**	ebi-a-GCST	13	-0.3145609	0.48287332	0.528140356	-0.1301388	0.13278577	0.32705378	-0.0349	0.12974	0.788079	20.407566	0.040054695	21.0813	0.04920203	0.018496817	0.031	0.558968
**60**	ebi-a-GCST	13	-0.16612465	0.28082241	0.566100847	0.21869879	0.12823906	0.08812001	0.22506	0.10061	0.025291	11.4656448	0.405116713	13.7554	0.31659835	0.028201235	0.019	0.16637
**61**	ebi-a-GCST	9	0.588512203	0.71518777	0.437707712	0.00570812	0.16017801	0.97157253	-0.1204	0.13804	0.382936	10.5587575	0.159057353	12.0982	0.14687663	-0.046863876	0.046	0.346021
**62**	ebi-a-GCST	13	-0.69476931	0.3605511	0.080198899	-0.2446752	0.12636035	0.05282746	-0.093	0.09596	0.33266	11.4037511	0.410084026	14.4792	0.27116025	0.046992298	0.027	0.112963
**63**	ebi-a-GCST	13	-0.43470222	0.23423537	0.090445861	-0.0945871	0.1182739	0.4238677	-0.0494	0.10207	0.628439	17.2989927	0.099337299	22.3662	0.03361451	0.033656835	0.019	0.100143
**64**	ebi-a-GCST	13	0.083833313	0.45613529	0.857521985	0.00389949	0.12747293	0.97559594	0.07457	0.08898	0.401998	12.441992	0.331352953	12.4425	0.4108291	-0.000685427	0.033	0.983823
**65**	ebi-a-GCST	13	0.083833313	0.45613529	0.857521985	0.00389949	0.12747293	0.97559594	0.07457	0.08898	0.401998	12.441992	0.331352953	12.4425	0.4108291	-0.000685427	0.033	0.983823
**66**	ebi-a-GCST	8	0.044936408	0.30068941	0.886099602	0.04345781	0.16771209	0.79554147	-0.0426	0.14388	0.767323	12.4686349	0.052294436	12.7072	0.07957291	-0.008762784	0.026	0.746305
**67**	ebi-a-GCST	13	-0.05687041	0.30740836	0.85659622	0.10946925	0.11135094	0.3255576	0.06941	0.08356	0.406148	12.5693542	0.322400841	12.7788	0.38532215	0.011256498	0.026	0.676826
**68**	ebi-a-GCST	15	0.288223342	0.16970062	0.113219054	0.07195753	0.05651535	0.20293336	0.04988	0.03936	0.20505	8.63297839	0.800063688	10.7177	0.70805972	-0.034999883	0.024	0.172448
**69**	ebi-a-GCST	11	0.71367196	0.35183781	0.073121819	0.03281854	0.08657518	0.70463168	-0.0387	0.07848	0.621562	10.4467564	0.315542035	15.9546	0.100939	-0.074607925	0.034	0.057331
**70**	ebi-a-GCST	4	-0.28800572	0.97482347	0.795504367	-0.0855455	0.09037224	0.34384775	-0.112	0.07778	0.149765	0.55430344	0.757939496	0.5871	0.89937883	0.022681966	0.125	0.872979
**71**	ebi-a-GCST	10	0.373196935	0.34111831	0.305781364	-0.0153593	0.14683318	0.91669012	-0.0291	0.10981	0.790735	6.13109932	0.632549492	7.68304	0.56637594	-0.031151283	0.025	0.248095
**72**	ebi-a-GCST	9	-0.16027943	0.26701704	0.567250227	-0.1106513	0.07449403	0.13744549	-0.0743	0.05524	0.17856	5.4392786	0.606517767	5.54759	0.69776203	0.011481021	0.035	0.751704
**73**	ebi-a-GCST	7	0.109519316	0.25903053	0.690005375	-0.0640799	0.12766448	0.61570992	0.03169	0.10059	0.752744	4.90449782	0.427646351	5.01081	0.54242725	-0.008410607	0.026	0.757585
**74**	ebi-a-GCST	9	-0.242363	0.42556755	0.58681541	0.04694751	0.15372136	0.76005654	0.05325	0.11388	0.640045	5.03569252	0.655607352	5.55543	0.69689101	0.021520004	0.03	0.494319
**75**	ebi-a-GCST	10	-0.28376417	0.26117661	0.308914287	-0.0093284	0.0913766	0.91868742	-0.0089	0.07043	0.898893	8.23970147	0.410412695	9.46745	0.39528904	0.030070448	0.028	0.306706
**76**	ebi-a-GCST	11	0.02052032	0.2997862	0.946924187	0.04522541	0.12660048	0.72092012	0.0261	0.11518	0.820711	14.9472399	0.092395438	14.9479	0.13397184	0.000442264	0.022	0.984194
**77**	ebi-a-GCST	8	0.247049287	0.81119846	0.77099688	-0.0389573	0.1301981	0.76477575	-0.0903	0.1027	0.379394	4.80047684	0.569646457	4.97622	0.66286589	-0.024352181	0.058	0.689655
**78**	ebi-a-GCST	9	0.081908883	0.90743484	0.930605781	0.06170786	0.16332974	0.70557061	0.07089	0.15182	0.640549	13.7165162	0.056459309	13.7168	0.08945174	-0.000662489	0.054	0.990496
**79**	ebi-a-GCST	9	-0.0268111	0.30229636	0.931811266	-0.092842	0.10019033	0.35410504	-0.1041	0.08354	0.212878	2.50040888	0.927066273	2.57111	0.95833221	-0.008038127	0.03	0.797974
**80**	ebi-a-GCST	10	-0.25723624	0.25090788	0.335256654	-0.1492687	0.10974118	0.17377017	-0.0793	0.07995	0.321476	7.93797682	0.439552119	8.49797	0.484843	0.01884032	0.025	0.475685
**81**	ebi-a-GCST	11	-0.94699687	0.3594252	0.027146259	0.08822756	0.13273264	0.50624153	-0.033	0.09501	0.728224	4.84580767	0.847537056	11.722	0.30408945	0.069293683	0.026	0.027708
**82**	ebi-a-GCST	10	0.785822731	0.53597575	0.18077812	0.03009556	0.15619341	0.84720851	-0.0169	0.20894	0.935556	23.4836161	0.002795782	31.0459	0.00029072	-0.05670936	0.035	0.147151
**83**	ebi-a-GCST	10	-0.06538579	0.33839374	0.851599085	-0.0531897	0.0864891	0.53856302	0.00081	0.0692	0.990712	11.1051878	0.195810058	11.1609	0.2648406	0.007439264	0.037	0.846206
**84**	ebi-a-GCST	11	0.253796118	0.44391419	0.581505012	-0.0521392	0.07626535	0.4941932	-0.0601	0.05995	0.316467	5.76701679	0.762990791	6.27617	0.79155175	-0.033082053	0.046	0.493594
**85**	ebi-a-GCST	6	0.413922288	0.28106713	0.214824931	-0.1591849	0.13382325	0.23423673	0.00571	0.12161	0.96256	5.04589354	0.282620865	8.15718	0.14778313	-0.057459543	0.037	0.191395
**86**	ebi-a-GCST	15	-0.18853761	0.29287819	0.530936918	-0.0931685	0.0941842	0.3225578	-0.0932	0.07228	0.197212	16.3916583	0.228625594	16.5346	0.2818305	0.007908699	0.023	0.741761
**87**	ebi-a-GCST	15	-0.18853761	0.29287819	0.530936918	-0.0931685	0.0941842	0.3225578	-0.0932	0.07228	0.197212	16.3916583	0.228625594	16.5346	0.2818305	0.007908699	0.023	0.741761
**88**	ebi-a-GCST	10	-0.20087056	0.30266568	0.525560604	0.03234204	0.12175104	0.79051586	0.16481	0.09701	0.089346	6.66967026	0.572655307	8.29653	0.50456099	0.028747828	0.023	0.237926
**89**	ebi-a-GCST	10	-0.00807274	0.2231539	0.972028683	-0.0254828	0.07020025	0.71660378	0.02167	0.05419	0.689164	8.39776927	0.39560993	8.41765	0.49266528	0.003852285	0.028	0.893936
**90**	ebi-a-GCST	13	-0.23782595	0.39146349	0.555833103	0.06556796	0.13469538	0.62640931	0.11657	0.10001	0.243785	6.35426718	0.848712153	7.23106	0.84197417	0.022590243	0.024	0.369185
**91**	ebi-a-GCST	6	1.247033876	0.74862837	0.17109113	0.19726814	0.20697875	0.3405475	0.3216	0.21304	0.131138	9.67128983	0.046344154	13.6514	0.01798217	-0.075681109	0.059	0.268778
**92**	ebi-a-GCST	9	0.375413633	0.34805502	0.316522562	0.05184295	0.08325162	0.5334646	0.02725	0.0672	0.685116	9.40347383	0.224971117	10.7995	0.21332321	-0.045829258	0.045	0.341945
**93**	ebi-a-GCST	16	0.132704108	0.15203376	0.397463837	-0.0120156	0.09713464	0.90155275	-0.0944	0.07364	0.200081	10.8129197	0.700666186	13.7271	0.54631537	-0.021235528	0.012	0.109873
**94**	ebi-a-GCST	11	-0.21225547	0.21699783	0.353561025	0.00730972	0.06841301	0.91491039	0.01994	0.04923	0.685443	9.57245139	0.386192244	10.8549	0.36893058	0.034575035	0.031	0.300674
**95**	ebi-a-GCST	5	-0.20515445	0.40273065	0.645571901	-0.1969009	0.11670615	0.09157423	-0.1578	0.09399	0.093117	4.70076439	0.195066533	4.7241	0.31679577	0.005279864	0.043	0.910593
**96**	ebi-a-GCST	8	-0.16892474	0.64195688	0.801245489	-0.1755786	0.16888405	0.29850716	-0.0673	0.163	0.679555	12.9886376	0.043216738	13.0472	0.07096568	0.006689931	0.041	0.874713
**97**	ebi-a-GCST	18	-0.1305832	0.17496856	0.4662971	-0.0754809	0.07327213	0.30294216	-0.1269	0.0523	0.015247	12.7320146	0.692241722	12.7325	0.75390126	0.000369567	0.017	0.982722
**98**	ebi-a-GCST	15	-0.02108843	0.37467019	0.955970412	-0.0654236	0.1095554	0.55039145	-0.0789	0.08386	0.346555	7.94179556	0.847371392	7.96689	0.89104367	-0.004437847	0.028	0.876559
**99**	ebi-a-GCST	9	-0.31353434	0.43991022	0.499079207	-0.1284023	0.12388747	0.29999564	-0.0664	0.141	0.637633	17.6527568	0.013638888	18.5487	0.01746884	0.021443191	0.036	0.569909
**100**	ebi-a-GCST	10	-0.098134	0.19119331	0.621632	-0.0667416	0.11826197	0.57251357	-0.04	0.09317	0.667617	10.5328767	0.229596698	10.698	0.29697924	0.007036187	0.02	0.732391
**101**	ebi-a-GCST	13	-0.41901046	0.41611239	0.335588993	0.20334017	0.11997392	0.09010014	0.08398	0.08798	0.339849	7.95826517	0.717037438	9.48778	0.66079909	0.041492407	0.034	0.24194
**102**	ebi-a-GCST	8	-0.24070871	0.3161146	0.475236515	-0.0452236	0.1287531	0.72540641	0.05516	0.10189	0.588287	6.01683079	0.421307328	6.99744	0.42914608	0.026222148	0.027	0.360911
**103**	ebi-a-GCST	5	0.125244331	0.47750265	0.810077177	-0.2328316	0.15679697	0.13756398	-0.1953	0.1197	0.102825	2.79078012	0.425019924	3.27154	0.51345442	-0.027147863	0.039	0.537939
**104**	ebi-a-GCST	18	0.132384724	0.31685113	0.681636666	-0.0326593	0.11825507	0.78241232	-0.1172	0.08406	0.163089	16.7037672	0.405011953	17.4019	0.42747696	-0.017469739	0.021	0.42552
**105**	ebi-a-GCST	12	0.114914071	0.20539058	0.588138283	-0.0084731	0.06493404	0.89618088	0.01558	0.04774	0.744166	9.67044427	0.46986632	9.91771	0.53780437	-0.015364864	0.031	0.62976
**106**	ebi-a-GCST	9	0.00467599	0.16305697	0.977922633	-0.1004468	0.09421361	0.286351	-0.0966	0.07281	0.184817	2.75204132	0.906954211	3.23348	0.91886214	-0.013255952	0.019	0.510132
**107**	ebi-a-GCST	11	0.139541526	0.2471681	0.586165453	0.18856463	0.09022574	0.03662493	0.08114	0.08229	0.324167	17.0616339	0.047759498	17.1822	0.07042753	-0.006798607	0.027	0.806531
**108**	ebi-a-GCST	14	-0.21118207	0.2289156	0.374427499	-0.1381947	0.09062915	0.12729978	-0.1391	0.07512	0.064065	16.0797441	0.187610882	16.2299	0.23693654	0.007400164	0.022	0.743569
**109**	ebi-a-GCST	9	0.496099389	0.2599832	0.098017067	0.09497218	0.0761053	0.21206589	0.04937	0.07365	0.502624	11.5125868	0.11777052	16.6963	0.03343125	-0.071523105	0.04	0.119102
**110**	ebi-a-GCST	5	0.406000876	0.47294812	0.453762751	-0.0280254	0.10307874	0.78571188	-0.006	0.07778	0.938782	3.01772734	0.388900037	3.80233	0.43341755	-0.054079448	0.061	0.442181
**111**	ebi-a-GCST	15	-0.41405736	0.31607617	0.212874856	-0.0672752	0.10067922	0.50399752	-0.0305	0.09908	0.758463	22.4523429	0.048737293	25.2602	0.03208414	0.032117664	0.025	0.22461
**112**	ebi-a-GCST	16	-0.26498623	0.19444468	0.19447002	-0.014836	0.0971036	0.87856783	0.02701	0.07037	0.701048	12.1771118	0.592081734	14.7721	0.46795334	0.026938353	0.017	0.12951
**113**	ebi-a-GCST	13	-0.03165608	0.38972982	0.936721509	-0.1378023	0.13439758	0.30520591	-0.1135	0.1101	0.302512	16.0199107	0.140393309	16.0903	0.18713674	-0.005742238	0.026	0.830061
**114**	ebi-a-GCST	12	-0.10808467	0.28298666	0.710494903	0.05774046	0.11663623	0.62056665	0.16076	0.107	0.132971	15.8754036	0.103255099	17.5459	0.09273243	0.021420276	0.021	0.329158
**115**	ebi-a-GCST	9	-0.07099413	0.28275172	0.808961002	0.01492528	0.08527301	0.86105661	0.01237	0.06959	0.858954	2.41450117	0.933396448	2.50703	0.9614025	0.009909587	0.033	0.769835
**116**	ebi-a-GCST	3	-0.14988214	3.32127802	0.971290196	-0.12521	0.22976169	0.58578385	-0.0793	0.19749	0.687857	0.81223226	0.367461104	0.81268	0.66608203	0.004153434	0.195	0.986458
**117**	ebi-a-GCST	15	-0.12558955	0.14991355	0.417309579	-0.0417624	0.11955479	0.72685191	-0.0335	0.07508	0.655758	9.67903655	0.719946359	10.1831	0.74868135	0.007906495	0.011	0.490279
**118**	ebi-a-GCST	13	0.303771033	0.37311105	0.432826683	-0.0684823	0.10726637	0.52319234	-0.0235	0.08549	0.783546	16.7821176	0.114487738	18.0219	0.11502655	-0.030625049	0.034	0.386653
**119**	ebi-a-GCST	12	-0.2352078	0.40755227	0.576617385	-0.0067964	0.12910388	0.95801652	0.05981	0.09571	0.532022	10.6514989	0.385305893	11.2437	0.42307955	0.01949782	0.026	0.473052
**120**	ebi-a-GCST	11	-0.10045162	0.32935286	0.767303996	-0.0297961	0.0849976	0.72592461	0.00934	0.0639	0.883816	6.64819179	0.673694304	6.76366	0.74755276	0.011485875	0.034	0.7418
**121**	ebi-a-GCST	10	-0.0832411	0.41285849	0.845245061	0.09091444	0.14491848	0.53043059	0.0423	0.12849	0.741971	15.0130804	0.058891619	15.2075	0.08539111	0.010174622	0.032	0.755786
**122**	ebi-a-GCST	10	-0.0832411	0.41285849	0.845245061	0.09091444	0.14491848	0.53043059	0.0423	0.12849	0.741971	15.0130804	0.058891619	15.2075	0.08539111	0.010174622	0.032	0.755786
**123**	ebi-a-GCST	9	0.282263887	0.21364804	0.22799352	0.06105267	0.09476443	0.51940867	0.02953	0.08424	0.725896	10.857743	0.1449457	13.3935	0.0990094	-0.032026471	0.025	0.241798
**124**	ebi-a-GCST	9	-0.22127063	0.25849941	0.420340377	-0.082022	0.07136968	0.25045082	-0.0942	0.05763	0.102071	0.90018428	0.996282126	1.15439	0.99707221	0.017198117	0.034	0.629605

**125**	ebi-a-GCST9	10	0.179335862	0.58662305	0.767630056	0.12146281	0.13650299	0.37356366	0.04991	0.14165	0.724551	20.0236947	0.010246777	20.154	0.01698535	-0.011504444	0.05	0.82523
**126**	ebi-a-GCST9	6	0.214284847	0.26450085	0.463298431	0.05318895	0.09088921	0.55840891	0.04889	0.07041	0.487466	1.34937239	0.852944846	1.77021	0.87993873	-0.024505951	0.038	0.551883
**127**	ebi-a-GCST9	7	-0.51230065	0.46384842	0.319688778	0.08376209	0.16765156	0.61734259	0.14882	0.16287	0.36085	7.96941057	0.157929548	11.5746	0.07216046	0.051585937	0.034	0.192921
**128**	ebi-a-GCST9	10	0.028190032	0.15857555	0.863322149	0.10035218	0.06903068	0.14602032	0.10391	0.04955	0.035989	7.12182459	0.523547662	7.37447	0.59819299	0.010821903	0.022	0.628761
**129**	ebi-a-GCST9	13	-0.26861912	0.3354754	0.440250353	0.11347151	0.10305342	0.27085574	0.12249	0.09037	0.175264	18.9775282	0.061498081	21.4986	0.04353852	0.038133344	0.032	0.252064
**130**	ebi-a-GCST9	8	-0.04198106	0.38965093	0.917714271	0.15448777	0.1146849	0.17795999	0.19461	0.09234	0.035065	2.87838615	0.823948618	3.26901	0.85905226	0.023437006	0.037	0.554982
**131**	ebi-a-GCST9	11	-0.19704962	0.25136008	0.453215811	-0.0785564	0.07050746	0.26521178	0.01251	0.05267	0.812246	9.59526757	0.384232287	10.3713	0.40854029	0.031963379	0.037	0.415684
**132**	ebi-a-GCST9	5	-0.98066652	0.98437864	0.392564466	0.2004905	0.18925833	0.28944116	0.01071	0.15893	0.946296	3.53496068	0.316256065	4.76173	0.31263017	0.087020014	0.085	0.382672
**133**	ebi-a-GCST9	13	0.099195234	0.22833526	0.672376583	-0.055481	0.08332994	0.50553943	-0.1019	0.05985	0.088787	12.0312704	0.36129732	12.9428	0.37321098	-0.022134067	0.024	0.380862
**134**	ebi-a-GCST9	14	0.104038197	0.19088654	0.595720018	0.03893983	0.09687477	0.6877131	0.04634	0.06871	0.499995	10.8360335	0.543020535	10.941	0.61576124	-0.005027307	0.016	0.751539
**135**	ebi-a-GCST9	12	-0.21512606	0.30238559	0.493079287	-0.0948129	0.0814832	0.24459101	-0.0242	0.07649	0.751316	20.4956445	0.024898304	21.3715	0.02972018	0.025795624	0.039	0.528042
**136**	ebi-a-GCST9	8	-0.00246529	0.47098993	0.995993368	-0.0419204	0.12518161	0.73771798	-0.0746	0.09678	0.441072	4.58745998	0.597702213	4.61192	0.70720111	-0.006140188	0.039	0.880839
**137**	ebi-a-GCST9	11	0.269186053	0.28501831	0.369601275	0.06067611	0.0649025	0.34984973	0.04424	0.04902	0.366875	5.71218329	0.768342703	6.35409	0.784689	-0.031855888	0.04	0.443659
**138**	ebi-a-GCST9	15	-0.31312732	0.18479198	0.11397905	-0.0479212	0.08797208	0.58593821	-0.0798	0.0633	0.207489	10.0946631	0.686175711	11.901	0.61425141	0.024388238	0.018	0.201933
**139**	ebi-a-GCST9	11	0.057100832	0.37730717	0.883047177	-0.053079	0.06592339	0.42072656	-0.0561	0.05776	0.33111	14.8301047	0.095709674	14.9825	0.13270276	-0.015920358	0.052	0.767988
**140**	ebi-a-GCST9	13	-0.10229298	0.32071096	0.755733993	0.06490074	0.12664576	0.6083299	0.07062	0.10739	0.510753	18.560647	0.069450549	19.1173	0.08573778	0.015095089	0.026	0.577289
**141**	ebi-a-GCST9	13	-0.18034814	0.37265304	0.637907252	-0.0027446	0.14094435	0.98446408	0.09961	0.12233	0.415474	18.1386417	0.078418886	19.1857	0.0841454	0.021532864	0.027	0.442404
**142**	ebi-a-GCST9	11	-0.54969212	0.43621902	0.239314897	0.17776874	0.14993001	0.23574943	0.06052	0.10434	0.561914	6.65566732	0.67291943	8.73126	0.55778415	0.039548606	0.027	0.183541
**143**	ebi-a-GCST9	15	-0.13001096	0.24135494	0.599217012	0.05370398	0.11836658	0.65003829	0.0825	0.09689	0.394455	18.9490466	0.124691968	20.2976	0.12103411	0.020027648	0.021	0.353668
**144**	ebi-a-GCST9	8	-0.48301409	0.65501646	0.488697296	-0.1043008	0.15922875	0.51244355	-0.1959	0.12866	0.127839	7.35226476	0.289494462	7.5984	0.36933391	0.018767695	0.042	0.669754
**145**	ebi-a-GCST9	13	-0.44484136	0.29206822	0.155957788	-0.0727667	0.12154544	0.54938694	-0.0338	0.08849	0.70211	8.08897627	0.705311977	10.2693	0.59234707	0.031453968	0.021	0.16783
**146**	ebi-a-GCST9	22	0.378473231	0.21629475	0.095481053	0.14765225	0.09933042	0.13715338	0.12925	0.083	0.119396	30.2011492	0.066659226	32.5418	0.0515514	-0.020607119	0.017	0.227521
**147**	ebi-a-GCST9	12	-0.17307691	0.33064567	0.612062313	0.03667161	0.11438279	0.74851033	0.05993	0.09953	0.547104	14.1198466	0.167594894	14.8942	0.18739229	0.018128099	0.024	0.475997
**148**	ebi-a-GCST9	11	0.489192174	0.43011731	0.284766439	0.01182625	0.10671656	0.91175966	-0.0351	0.07745	0.650368	6.41147092	0.69813409	7.94713	0.6340009	-0.044724506	0.036	0.246606
**149**	ebi-a-GCST9	14	-0.10319079	0.2228905	0.651671874	-0.189984	0.10974164	0.08341728	-0.1122	0.07953	0.158132	12.7744762	0.385643666	12.7765	0.46521921	-0.000818174	0.019	0.965855
**150**	ebi-a-GCST9	12	0.315612167	0.25828842	0.249753186	0.05285999	0.08892322	0.55221431	-0.0382	0.06459	0.553732	8.59903243	0.570531617	10.6012	0.47725406	-0.035684467	0.025	0.187453
**151**	ebi-a-GCST9	6	0.309013094	0.33677434	0.410764668	0.09022637	0.15011163	0.54779883	-0.0261	0.12407	0.833132	4.73547742	0.315531363	6.08629	0.29791322	-0.025521241	0.024	0.345617
**152**	ebi-a-GCST9	8	0.01320075	0.27062081	0.962678233	0.05258921	0.0719359	0.46474461	0.06239	0.05426	0.250224	4.47002564	0.613340995	4.50445	0.72018104	0.006654823	0.036	0.858915
**153**	ebi-a-GCST9	12	0.248534878	0.2654501	0.37119014	0.04122522	0.12392855	0.73939614	-0.0595	0.09565	0.533957	10.0120078	0.439440433	11.5507	0.39834738	-0.024141895	0.019	0.243396
**154**	ebi-a-GCST9	11	0.16416853	0.26933868	0.55724924	-0.0168081	0.1276219	0.89521979	0.00153	0.11081	0.989003	16.1500651	0.063813201	16.9473	0.07553901	-0.01541479	0.023	0.521792
**155**	ebi-a-GCST9	10	-0.03570906	0.3270867	0.915753989	-0.0674385	0.1436527	0.63874412	-0.0154	0.11558	0.894191	12.9401896	0.113924135	12.9475	0.16499793	0.001694187	0.025	0.94818
**156**	ebi-a-GCST9	15	0.283003853	0.19974814	0.180056718	0.09211285	0.11474162	0.42209904	0.04739	0.08474	0.576006	16.2697241	0.234871183	18.3719	0.1903658	-0.020630636	0.016	0.217507
**157**	ebi-a-GCST9	12	0.152611541	0.42222254	0.725284509	0.01000261	0.12445908	0.93594402	0.01303	0.09759	0.893785	13.9483311	0.175361462	14.1102	0.2269571	-0.010275598	0.03	0.74043
**158**	ebi-a-GCST9	12	-0.05694277	0.34885785	0.873591809	-0.0756354	0.08378157	0.36664872	-0.0781	0.07052	0.268056	14.985193	0.132602627	14.991	0.18291094	-0.002310509	0.037	0.951736
**159**	ebi-a-GCST9	9	-0.13437508	0.43475315	0.76625456	0.01450907	0.18096542	0.93609733	-0.0985	0.12711	0.438309	8.53500502	0.287785474	8.5442	0.38219159	0.002309293	0.027	0.933236
**160**	ebi-a-GCST9	9	-0.21962393	0.27968892	0.458059344	-0.0244859	0.06281485	0.6966769	-0.0129	0.04787	0.786888	5.22268941	0.632807834	5.78524	0.67127332	0.030292873	0.04	0.477679
**161**	ebi-a-GCST9	5	-0.2107113	0.41276427	0.644899822	-0.0839529	0.12321811	0.4956593	-0.0928	0.10684	0.384866	0.21918765	0.974433885	0.30659	0.98938485	0.011015112	0.037	0.786788
**162**	ebi-a-GCST9	6	0.724667715	0.6905494	0.353209476	0.04222566	0.12016728	0.72529527	0.08713	0.1051	0.407083	5.80412469	0.214261698	7.07102	0.21541172	-0.064181956	0.069	0.402997
**163**	ebi-a-GCST9	14	0.47166527	0.48272904	0.347816251	0.08373565	0.14069415	0.55173595	0.12442	0.13079	0.341439	25.8447266	0.011290021	27.0508	0.0122434	-0.027173215	0.036	0.468681
**164**	ebi-a-GCST9	11	-0.37867933	0.25815447	0.176463406	-0.200296	0.13289255	0.13175866	-0.1516	0.09917	0.126355	9.80115887	0.366821201	10.7913	0.37401045	0.017985523	0.019	0.365241
**165**	ebi-a-GCST9	12	-0.46575335	0.42644559	0.300369455	-0.1404361	0.12564577	0.26368894	-0.0241	0.099	0.807512	12.7586083	0.237483218	14.2039	0.22191382	0.030352011	0.029	0.312199
**166**	ebi-a-GCST9	5	0.044338158	0.30308239	0.892969059	-0.2142461	0.12789317	0.09389581	-0.1865	0.10076	0.064122	1.64641439	0.648912052	2.2988	0.6809883	-0.024255548	0.03	0.478366
**167**	ebi-a-GCST9	10	0.200147409	0.32897527	0.559797582	-0.001767	0.10114226	0.98606161	-0.0109	0.07659	0.887224	4.38544836	0.820781231	4.82044	0.84967151	-0.022774251	0.035	0.528075
**168**	ebi-a-GCST9	13	-0.06330052	0.36162771	0.864225963	0.02885834	0.07524014	0.70131213	0.00754	0.06044	0.900676	14.7738286	0.193087077	14.827	0.25103467	0.010164859	0.051	0.845933
**169**	ebi-a-GCST9	15	0.003032463	0.18489829	0.987163753	-0.0918587	0.09434557	0.33023498	-0.0853	0.06753	0.206671	12.0683116	0.522050415	12.3315	0.57969613	-0.008636546	0.017	0.616516
**170**	ebi-a-GCST9	9	-0.07032284	0.26325841	0.797071896	-0.0360438	0.10046934	0.71977892	-0.0743	0.08236	0.36713	11.7478464	0.109161987	11.7483	0.16279363	-0.000478021	0.03	0.987721
**171**	ebi-a-GCST9	13	-0.23575237	0.25108874	0.367931191	-0.0066396	0.10178803	0.94799078	0.03358	0.08041	0.676248	12.8814523	0.301144231	14.3789	0.2771671	0.0231698	0.02	0.282181
**172**	ebi-a-GCST9	8	0.066499303	0.51653155	0.901768858	0.08799763	0.1212386	0.46794794	0.07684	0.11882	0.517833	15.9542385	0.014001707	15.9554	0.02552721	0.001047655	0.051	0.984177
**173**	ebi-a-GCST9	13	-0.13807559	0.21928794	0.541777957	0.05775626	0.07300009	0.42883855	0.05718	0.05134	0.265427	9.72879578	0.554934609	10.5676	0.56629338	0.02310492	0.025	0.379381
**174**	ebi-a-GCST9	12	-0.45812007	0.33661318	0.203403379	-0.0999445	0.10209285	0.32760106	-0.1599	0.09225	0.083047	15.2286643	0.123944897	16.5226	0.12281264	0.027583071	0.03	0.378354
**175**	ebi-a-GCST9	10	-0.41749648	0.27196664	0.163306959	0.05927553	0.11608096	0.60960347	0.07263	0.1116	0.515205	13.0233276	0.1110495	19.0948	0.02439867	0.05442796	0.028	0.089552
**176**	ebi-a-GCST9	12	0.151034595	0.22258458	0.5128197	0.14382184	0.09300517	0.1220115	0.0774	0.06842	0.257976	10.1177009	0.430228423	10.2401	0.50892508	-0.007215904	0.021	0.735153
**177**	ebi-a-GCST9	15	0.193641567	0.4621525	0.682056976	-0.0795088	0.10717506	0.45817279	-0.0906	0.08969	0.31237	18.540794	0.138044021	19.1024	0.16107224	-0.022946315	0.037	0.541178
**178**	ebi-a-GCST9	13	0.117623685	0.2342949	0.625541542	0.00759854	0.08097591	0.92523864	-0.0084	0.06264	0.893072	5.15190673	0.923587037	5.46359	0.94067856	-0.012872659	0.023	0.587831
**179**	ebi-a-GCST9	14	-0.00388353	0.21854459	0.986114401	-0.0074975	0.10194597	0.94137346	0.04701	0.07509	0.531298	7.36196066	0.832796496	7.42345	0.879032	0.004867942	0.02	0.808347
**180**	ebi-a-GCST9	11	-0.21861505	0.30337925	0.489448149	-0.0504428	0.07772917	0.51636724	-0.0197	0.05747	0.732244	10.3321457	0.324271303	10.8453	0.36969328	0.022817975	0.034	0.520526
**181**	ebi-a-GCST9	6	0.331426738	1.00662723	0.758488913	0.05502093	0.13105499	0.67460904	0.12143	0.11438	0.288386	5.84128796	0.211318658	5.90587	0.31548632	-0.016076399	0.076	0.843716
**182**	ebi-a-GCST9	10	0.065581686	0.45011022	0.887761406	0.02674062	0.07354239	0.71615055	-0.0032	0.05581	0.954458	11.0159136	0.200799142	11.0486	0.27239103	-0.009061066	0.059	0.881335
**183**	ebi-a-GCST9	5	0.042802326	0.29178338	0.892678198	-0.02925	0.1226414	0.81149335	0.03688	0.11106	0.739806	5.21640865	0.156619404	5.2173	0.26571934	-0.000705764	0.031	0.9834
**184**	ebi-a-GCST9	9	-0.02546239	0.21236589	0.907932094	0.0904972	0.06348144	0.15399263	0.10064	0.04943	0.041746	2.38664354	0.935388361	2.75945	0.94853474	0.019534197	0.032	0.560771
**185**	ebi-a-GCST9	14	0.006872115	0.17934395	0.970064178	-0.0470065	0.12252136	0.70123093	0.01736	0.08811	0.843808	7.09692517	0.851142373	7.10143	0.8968489	0.000950705	0.014	0.947581
**186**	ebi-a-GCST9	12	-0.57671968	0.28615451	0.071525753	-0.0211065	0.12481995	0.86572129	-0.0504	0.09716	0.603658	11.3899298	0.327956358	15.6347	0.15524487	0.040741954	0.021	0.082369
**187**	ebi-a-GCST9	10	-0.27412998	0.3398062	0.443145276	-0.0286549	0.14790192	0.84637771	0.11114	0.1099	0.311888	8.31856609	0.402986406	9.80354	0.3666227	0.028299488	0.024	0.266296
**188**	ebi-a-GCST9	13	0.073790153	0.32124909	0.822542064	-0.087937	0.12830553	0.49310949	-0.106	0.11508	0.356831	18.1804696	0.077486445	18.7795	0.09398954	-0.013516825	0.022	0.559346
**189**	ebi-a-GCST9	13	0.184344395	0.56779246	0.751522562	-0.149476	0.14666686	0.30813028	-0.0094	0.12864	0.941593	20.9415474	0.033981723	21.1763	0.04785812	-0.014299089	0.041	0.732125
**190**	ebi-a-GCST9	12	0.077555457	0.26654248	0.777020927	0.0627613	0.13523145	0.64257406	0.03444	0.10095	0.732968	14.6284984	0.146204523	14.6739	0.19791706	-0.003438875	0.02	0.863649
**191**	ebi-a-GCST9	7	0.133034447	0.72201874	0.861054455	-0.019872	0.14921982	0.8940566	-0.0059	0.12091	0.961151	2.83833552	0.724892732	2.87643	0.82418932	-0.010264049	0.053	0.852946
**192**	ebi-a-GCST9	13	-0.47733768	0.43787345	0.298961548	-0.0680927	0.13682386	0.61871866	-0.1489	0.101	0.140427	6.89088111	0.80785005	7.48513	0.82396196	0.020479559	0.027	0.457019
**193**	ebi-a-GCST9	9	-0.07032284	0.26325841	0.797071896	-0.0360438	0.10182628	0.72335873	-0.0743	0.08236	0.36713	11.7478464	0.109161987	11.7483	0.16279363	-0.000478021	0.03	0.987721
**194**	ebi-a-GCST9	15	-0.02623254	0.22530115	0.909088689	0.03490981	0.11406382	0.75956272	0.01885	0.08714	0.828715	7.71421609	0.861707274	7.7613	0.9013871	0.003566503	0.016	0.83158
**195**	ebi-a-GCST9	10	-0.00194738	0.21780983	0.993085343	0.03586008	0.06883713	0.60240794	0.00572	0.05292	0.913884	5.70097812	0.680685972	5.7023	0.76930427	0.00127566	0.035	0.971931
**196**	ebi-a-GCST9	13	-0.23575237	0.25108874	0.367931191	-0.0066396	0.10289146	0.94854776	0.03358	0.08041	0.676248	12.8814523	0.301144231	14.3789	0.2771671	0.0231698	0.02	0.282181
**197**	ebi-a-GCST9	13	-0.13807559	0.21928794	0.541777957	0.05775626	0.07187024	0.4216173	0.05718	0.05134	0.265427	9.72879578	0.554934609	10.5676	0.56629338	0.02310492	0.025	0.379381
**198**	ebi-a-GCST9	14	0.081207227	0.13209594	0.550193077	0.01683061	0.08849029	0.84915458	0.00221	0.06073	0.970925	11.832524	0.459221975	12.286	0.50434595	-0.010457521	0.016	0.513467
**199**	ebi-a-GCST9	14	0.02037508	0.25321744	0.937194024	-0.0030419	0.08049247	0.96985425	-0.0452	0.06249	0.469811	5.46397194	0.940662892	5.53531	0.96144536	-0.006596943	0.025	0.793939
**200**	ebi-a-GCST9	12	-0.02519169	0.5585548	0.964914299	0.23210036	0.15866476	0.14351253	0.09391	0.15469	0.543801	25.795766	0.0040248	25.9238	0.00666146	0.007863873	0.035	0.828184
**201**	ebi-a-GCST9	11	-0.23628501	0.28369711	0.426462538	0.06110554	0.109729	0.57761158	0.10912	0.08416	0.194793	3.12339598	0.959200939	4.7488	0.90731443	0.028395139	0.022	0.234268
**202**	ebi-a-GCST9	8	-0.29128274	0.22878541	0.250059394	-0.0142538	0.08224877	0.86241444	-0.0374	0.06436	0.561665	4.36436164	0.627495617	5.70212	0.57492895	0.037657022	0.033	0.291395
**203**	ebi-a-GCST9	15	-0.69224845	0.40712731	0.112853542	0.01224251	0.13374279	0.92706535	-0.0577	0.10121	0.56835	15.3960809	0.283282824	18.4419	0.18738711	0.039479302	0.025	0.13279
**204**	ebi-a-GCST9	11	-0.07225242	0.23571425	0.76617748	-0.054009	0.14009168	0.6998477	0.0326	0.1037	0.753244	6.97529063	0.639692123	7.22065	0.70446324	0.008300213	0.017	0.632232
**205**	ebi-a-GCST9	8	0.379054834	0.27614844	0.218958091	0.16931779	0.09983068	0.08987647	0.10588	0.07745	0.171607	2.92121138	0.818668722	3.98332	0.78169664	-0.033099154	0.032	0.342476
**206**	ebi-a-GCST9	12	0.214943665	0.20907463	0.32814113	0.02859398	0.06419904	0.65603372	0.00072	0.04718	0.98777	9.55559682	0.480307511	10.6618	0.47201951	-0.030959201	0.029	0.317669
**207**	ebi-a-GCST9	15	-0.29753532	0.21560809	0.190864011	-0.0400407	0.11999585	0.73861812	-0.0073	0.09622	0.939287	16.2441455	0.236197132	19.0157	0.16434898	0.024858909	0.017	0.160259
**208**	ebi-a-GCST9	9	-0.29187448	0.22939004	0.243866174	0.01309422	0.07989213	0.86981091	-0.0297	0.06027	0.622047	4.42564925	0.729650873	5.82866	0.66641762	0.039266406	0.033	0.274874
**209**	ebi-a-GCST9	12	0.434750016	0.33511698	0.223654763	0.06071974	0.14538778	0.67621041	-0.0255	0.12644	0.840209	15.5407367	0.113555826	18.9051	0.06281867	-0.032479703	0.022	0.171953
**210**	ebi-a-GCST9	12	-0.42869172	0.27329965	0.147817763	-0.0801239	0.11176093	0.47342223	-0.0888	0.08207	0.279502	9.87193689	0.451799736	11.5642	0.39727496	0.030986029	0.024	0.222479
**211**	ebi-a-GCST9	12	0.003646676	0.20990146	0.986480573	0.06226809	0.11075022	0.57395318	0.07108	0.07994	0.373976	9.28660008	0.505137405	9.4073	0.58435094	0.006419301	0.018	0.735474
