# Supplementary material for: Assessing the relationship between gut microbiota and endometriosis: a bidirectional two-sample mendelian randomization analysis
Source: BMC Womens Health. 2024 Feb 16;24:123. doi: 10.1186/s12905-024-02945-z (PMC10873948; doi:10.1186/s12905-024-02945-z)
Supplement: Supplementary file 3 — Supplementary Material 3 [file 12905_2024_2945_MOESM3_ESM.pdf]

Supplementary File 3. Analysis results of all SNPs related to these seven gut microbiota

exposure	SNP	effect_allele	other_allele	beta.exposure	beta.outcome	eaf.exposure	eaf.outcome	chr	pos	se.outcome	pval.outcome	se.exposure	pval.exposure	R2	F
class Melainabacteria	rs10148250	A	G	-0.0861928	0.0334	0.5437	0.5833	14	107061448	0.0186	0.0729407	0.0193381	8.67142E-06	0.001386735	19.86340308
	rs10738747	A	G	-0.0814624	0.0191	0.504	0.566	9	26184578	0.0177	0.2826	0.0184409	9.95795E-06	0.001362197	19.51144603
	rs11150282	T	C	0.0989487	-0.0253	0.3211	0.3599	16	80493705	0.0182	0.1661	0.0196972	6.02893E-07	0.001760871	25.23193034
	rs113884518	T	C	-0.205352	-0.011	0.0487	0.02384	9	24648997	0.0576	0.8493	0.0454982	8.05675E-06	0.001421914	20.36801583
	rs28678345	T	C	0.214848	-0.0926	0.0467	0.04881	17	53906328	0.0411	0.0242198	0.0470878	6.69083E-06	0.001453102	20.81541677
	rs367480	A	G	0.083758	-0.0212	0.5726	0.6419	11	2937631	0.0184	0.2484	0.0185796	8.19917E-06	0.001418552	20.31978733
	rs4129395	G	A	0.0896187	-0.0226	0.4264	0.4877	9	115975389	0.0176	0.1989	0.0185106	1.48057E-06	0.001635788	23.43665182
	rs789069	A	C	-0.103539	-0.0134	0.172	0.1417	18	1008278	0.0251	0.5943	0.0234287	6.84636E-06	0.00136333	19.52768812
	rs79790072	T	C	0.226722	-0.0045	0.0567	0.03149	15	100747683	0.0504	0.9292	0.0487841	3.29011E-06	0.0015075	21.59583567
	rs9864379	T	C	-0.159735	-0.0261	0.1133	0.1494	3	14306949	0.0247	0.2909	0.0292553	5.36056E-08	0.002079547	29.80782865
family Prevotellaceae	rs12057990	C	T	0.0589878	0.0008	0.2406	0.2677	1	99491816	0.0198	0.9675	0.0132268	8.9747E-06	0.001388332	19.88630544
	rs12118202	T	C	-0.0751986	-0.0035	0.1899	0.1807	1	210681370	0.0228	0.8796	0.0147265	5.5423E-07	0.00181933	26.07112115
	rs13069367	A	C	-0.0539768	-0.0123	0.3549	0.4101	3	71801830	0.0178	0.489	0.0119687	7.39486E-06	0.001419662	20.33572152
	rs148376875	T	G	0.084515	0.0477	0.1203	0.1532	3	170126707	0.0244	0.0510199	0.0179163	2.08027E-06	0.001553022	22.24898589
	rs2206482	T	G	-0.0569105	-0.0206	0.4423	0.3976	20	9771109	0.0179	0.249	0.0117486	1.30377E-06	0.001637505	23.46128783
	rs2278540	G	A	0.0554249	0.001	0.326	0.3641	3	32408916	0.0182	0.9544	0.0123265	8.43587E-06	0.001411233	20.21480504
	rs34660375	A	G	-0.0805894	0.0296	0.1909	0.1538	5	180283881	0.025	0.2372	0.0178291	7.40321E-06	0.00142613	20.42849082
	rs3758087	C	T	-0.0564199	-0.0261	0.7078	0.722	8	23714609	0.0196	0.1821	0.0124655	8.60578E-06	0.0014299	20.48257241
	rs3860225	A	G	0.083936	0.0983	0.1382	0.08994	1	111161677	0.0307	0.00135101	0.0167936	5.4968E-07	0.001743143	24.97745638
	rs4493272	T	C	-0.0604992	0.0032	0.4712	0.4777	2	118910912	0.0176	0.8575	0.0118027	3.02315E-07	0.001833249	26.2709518
	rs4685827	T	C	-0.0679529	-0.0167	0.2237	0.2413	3	4864877	0.0206	0.4171	0.0145407	2.77192E-06	0.00152428	21.83658113
	rs7252711	G	A	0.0740876	0.0172	0.8728	0.8905	19	17460999	0.0281	0.541101	0.0161926	5.56583E-06	0.001461183	20.93134606
	rs7975087	C	A	-0.0601448	-0.0139	0.2286	0.1755	12	21404832	0.0231	0.546799	0.0135454	7.59451E-06	0.001376246	19.71295734
	rs912860	A	G	0.229046	0.0152	0.9732	0.9769	14	33706908	0.0581	0.7935	0.0483124	9.29929E-07	0.001568658	22.47333262
	rs9586501	G	A	0.0592681	-0.0005	0.3141	0.2638	13	105075823	0.0199	0.9804	0.0127329	2.58988E-06	0.001512209	21.66340077
	rs9958960	G	A	-0.0914807	-0.0026	0.165	0.1545	18	63018983	0.0242	0.9144	0.0173611	1.06324E-07	0.001937065	27.76155329
genus Anaerotruncus	rs10150232	A	G	0.0567088	0.0096	0.2535	0.1992	14	30418008	0.022	0.6633	0.0124877	6.67667E-06	0.001439435	20.61936478
	rs11018566	A	G	-0.156465	-0.0361	0.0517	0.05284	11	89040226	0.0392	0.3563	0.0366032	6.13705E-06	0.001275627	18.26987517
	rs115414803	A	C	-0.144356	-0.0167	0.0586	0.0623	4	88163243	0.0367	0.6489	0.0317524	6.83471E-06	0.001442684	20.66597245
	rs1272208	T	G	0.0611743	0.006	0.7197	0.7607	9	78630894	0.0207	0.773101	0.0129831	4.27986E-06	0.001549493	22.19833972
	rs1431492	C	T	-0.0654996	-0.0035	0.1829	0.1571	3	150855371	0.0239	0.8845	0.0146188	7.35563E-06	0.001401286	20.07211457
	rs17734739	T	C	0.0660052	0.0144	0.1899	0.1414	2	211663702	0.0251	0.5672	0.014908	7.4278E-06	0.001368373	19.60003357
	rs34449434	A	C	-0.0497004	-0.0142	0.4563	0.3909	12	76523655	0.0185	0.4427	0.0113402	9.85054E-06	0.001340841	19.20514634
	rs4669806	G	T	0.0576389	0.0157	0.2495	0.2146	2	12200752	0.0213	0.4622	0.0122994	2.42058E-06	0.001532777	21.95850643
	rs6494922	A	G	0.0903106	-0.0772	0.0726	0.05324	15	33459867	0.0394	0.0499195	0.0202257	6.62369E-06	0.001391705	19.93469583
	rs6563550	T	C	0.0877135	0.0034	0.0785	0.07785	13	38058413	0.0327	0.9184	0.0176745	2.34515E-07	0.001718594	24.62509355
	rs7155595	C	A	0.0539336	0.0572	0.3091	0.3008	14	77502546	0.0192	0.002852	0.0118903	7.54988E-06	0.00143612	20.57180864
	rs8005030	C	T	0.0554447	0.0185	0.3022	0.3331	14	30607199	0.0186	0.3203	0.0117854	2.28297E-06	0.001544692	22.12945418
	rs9347879	T	C	0.050618	0.0303	0.4602	0.4895	6	165015261	0.0176	0.0839499	0.011049	4.22328E-06	0.001464904	20.98472569
genus Eubacterium ruminantium group	rs10131724	C	A	0.199832	-0.0162	0.9364	0.9008	14	52219066	0.0297	0.586001	0.0414577	2.38666E-06	0.001621424	23.23052055
	rs10923018	G	A	0.0726438	0.005	0.4722	0.5786	1	88523399	0.0177	0.7758	0.0160924	6.79548E-06	0.001422391	20.37486551
	rs11637981	T	G	0.073258	-0.0277	0.5159	0.4417	15	61289622	0.0177	0.1174	0.0160888	5.43676E-06	0.001447157	20.7301331
	rs13025464	C	T	0.0737077	-0.0273	0.6173	0.5985	2	201610645	0.0179	0.1279	0.0163786	6.96835E-06	0.001413642	20.24936676
	rs139749	C	T	-0.0845439	-0.0195	0.3419	0.3403	22	25303055	0.0186	0.2959	0.0171791	8.58836E-07	0.001690094	24.21603419
	rs16891896	G	A	-0.174787	-0.0004	0.0586	0.08453	5	33897589	0.0315	0.9908	0.0390573	2.38122E-06	0.001397938	20.02410446
	rs17519472	C	T	0.107804	-0.0037	0.1541	0.1402	12	30360954	0.0254	0.8853	0.0233984	4.7036E-06	0.001481615	21.22446292
	rs209813	G	A	-0.103488	0.0055	0.166	0.1447	6	11953633	0.0249	0.8263	0.0236391	9.23145E-06	0.001337884	19.16273054
	rs2116427	A	G	0.0911461	-0.0104	0.2565	0.255	5	121182996	0.0202	0.606599	0.0182353	4.67369E-07	0.001743309	24.97983911
	rs2229917	A	G	0.153538	-0.0493	0.0666	0.04207	9	130980937	0.0438	0.2606	0.0323922	2.15894E-06	0.001568018	22.46415818
	rs2418654	C	T	-0.0748879	0.0054	0.4781	0.4302	2	71247578	0.018	0.7643	0.0165852	6.17248E-06	0.001423132	20.38549021
	rs2817174	C	T	-0.0734306	-0.0022	0.4294	0.3956	1	3044181	0.018	0.9011	0.0163687	7.86955E-06	0.001404744	20.1217179
	rs57340348	T	C	-0.0979429	0.0211	0.2147	0.2025	6	130030114	0.022	0.3355	0.0212166	4.93276E-06	0.001487406	21.30755516
	rs606117	G	A	-0.0833243	0.0179	0.668	0.7299	9	2539358	0.0198	0.3643	0.018056	4.81637E-06	0.001486401	21.29313151
	rs6676699	T	G	0.0888124	0.0009	0.7783	0.6969	1	197978281	0.0191	0.9632	0.0196447	6.37984E-06	0.001426652	20.43599046
	rs7000472	G	A	0.0762282	-0.0265	0.6382	0.5847	8	138626268	0.0179	0.1389	0.016523	4.06846E-06	0.001485558	21.28103787
	rs72836424	C	T	-0.139825	0.0731	0.0954	0.1109	10	129037453	0.0282	0.00952204	0.030069	2.62126E-06	0.001509237	21.62076053
	rs73139629	A	C	-0.115098	0.0032	0.1322	0.09146	12	63306003	0.0306	0.9155	0.0247909	5.36204E-06	0.001504454	21.55213248
genus Olsenella	rs1035588	A	G	-0.108148	-0.0416	0.3827	0.3756	2	150077190	0.0182	0.0221401	0.0236848	4.85501E-06	0.001455281	20.84667625
	rs17148768	G	A	0.140434	0.0011	0.2117	0.1684	10	10777085	0.0236	0.9636	0.0295602	2.19842E-06	0.001575168	22.56675307
	rs2759329	A	G	0.111131	-0.0108	0.66	0.6305	1	231960352	0.0182	0.5531	0.0237218	3.43202E-06	0.001531762	21.94393182
	rs35225860	A	G	-0.223604	-0.035	0.0567	0.03902	1	247642270	0.045	0.4366	0.0482391	3.87044E-06	0.001499653	21.48325207
	rs61090148	A	G	-0.104783	-0.0155	0.4225	0.4303	4	174810498	0.0178	0.3822	0.0231343	6.43724E-06	0.001431952	20.51200961
	rs62112538	C	T	-0.19943	-0.0543	0.0805	0.1115	19	4925018	0.0282	0.0544001	0.0407033	1.1882E-06	0.001675234	24.00275087
	rs72691585	C	A	-0.249079	0.0022	0.0686	0.1338	9	21884425	0.0257	0.9318	0.0520814	2.95291E-06	0.001596235	22.86905441
	rs7540303	C	T	0.108044	0.0112	0.4215	0.3648	1	179629191	0.0182	0.5389	0.023638	5.32023E-06	0.001458236	20.88907132
	rs8066522	A	G	0.106529	0.0128	0.6531	0.6697	17	59632873	0.0188	0.495801	0.0240379	9.70085E-06	0.001370972	19.63730715
	rs9460691	C	A	0.119966	0.0117	0.2167	0.1956	6	10422207	0.0224	0.6017	0.0268645	7.2835E-06	0.001391989	19.93875853
genus Oscillospira	rs12206468	G	A	-0.133016	-0.0038	0.0636	0.07733	6	18093691	0.0329	0.9086	0.0269733	1.04222E-06	0.001697007	24.31525805
	rs12925026	T	C	0.13559	0.056	0.0726	0.05635	16	89792856	0.0379	0.1399	0.030678	9.31022E-06	0.001363611	19.53172118
	rs1954532	C	T	0.0826246	0.0123	0.7913	0.8004	14	28151415	0.0221	0.579399	0.0175252	2.26772E-06	0.001551316	22.22450286
	rs28889936	A	C	0.114051	0.0142	0.1083	0.0961	4	89483300	0.0297	0.632	0.0252838	3.36589E-06	0.001420293	20.34476994
	rs62422654	C	T	0.0898456	0.0388	0.1471	0.2086	6	170474630	0.0216	0.0717695	0.0198088	6.46755E-06	0.001435936	20.56916079
	rs72866977	A	C	-0.130569	0.0061	0.0696	0.07952	6	56160483	0.0327	0.8522	0.0281674	5.62704E-06	0.001499742	21.48453282
	rs751183	C	T	0.0774322	0.0253	0.7545	0.8201	1	76803093	0.0228	0.2678	0.0172216	6.85125E-06	0.001411122	20.21321904
	rs8076323	A	G	0.0715392	0.013	0.2575	0.3258	17	14881860	0.0187	0.4869	0.0156539	5.61319E-06	0.001457778	20.88249225
order Bacillales	rs10233278	T	C	-0.116319	-0.0293	0.4125	0.556	7	117496144	0.0178	0.0992796	0.0248527	0.000003508	0.001528873	21.90248773
	rs10410917	C	T	-0.11471	-0.0162	0.4036	0.39	19	14970441	0.018	0.3669	0.0250025	5.57353E-06	0.001469193	21.0462618
	rs11034576	A	G	0.205765	-0.0026	0.0795	0.1237	11	38085711	0.0265	0.9206	0.0453311	8.85972E-06	0.00143816	20.60106984
	rs11207728	A	G	0.14455	0.0074	0.7972	0.8365	1	61827534	0.0238	0.756599	0.0316912	5.73456E-06	0.001452143	20.8016653
	rs11844714	A	G	-0.143245	-0.0132	0.163	0.2076	14	49001226	0.0217	0.5439	0.0320272	5.06209E-06	0.001396355	20.00139472
	rs1287018	G	A	0.141125	0.0213	0.16	0.1881	20	5826474	0.0225	0.3442	0.0319309	9.87097E-06	0.001363562	19.53101759
	rs4617108	G	A	-0.248861	-0.0419	0.9394	0.9142	7	49423832	0.0313	0.1818	0.0525929	1.97506E-06	0.001562651	22.38714577
	rs62640857	A	G	0.148238	0.0098	0.1839	0.2262	12	69344114	0.021	0.6402	0.0326455	4.49262E-06	0.001439225	20.61634747
	rs74420793	A	G	-0.164413	0.0016	0.162	0.1042	4	127766709	0.0287	0.9551	0.0353763	3.07319E-06	0.001507558	21.596669
