# Supplementary material for: Assessing the relationship between gut microbiota and endometriosis: a bidirectional two-sample mendelian randomization analysis
Source: BMC Womens Health. 2024 Feb 16;24:123. doi: 10.1186/s12905-024-02945-z (PMC10873948; doi:10.1186/s12905-024-02945-z)
Supplement: Supplementary file 4 — Supplementary Material 4 [file 12905_2024_2945_MOESM4_ESM.pdf]

Supplementary File 4. All SNP analysis results of EMs by seven gut microbiota

outcome	SNP	effect_allel	other_allel	beta.expos	beta.outcome	se.exposur	se.outcome	pval.expos	pval.outcom	chr	pos	samplesize	EAF2	MAF
class Melainabacteria	rs10757277	G	A	-0.1013	-0.00370104	0.0178	0.0191921	1.25E-08	0.821437	9	22124450	14306	0.5797	0.4203
	rs10917151	A	G	0.1685	0.0113619	0.0232	0.0249446	4.03E-13	0.710549	1	22422721	14306	0.8294	0.1706
	rs11031005	C	T	-0.2379	-0.00619805	0.0239	0.0261819	2.63E-23	0.837219	11	30226356	14306	0.8324	0.1676
	rs1566646	T	G	-0.1116	0.00425538	0.0225	0.0221753	6.80E-07	0.826553	12	115117029	14306	0.8069	0.1931
	rs17694933	A	G	0.1331	-0.0099531	0.0177	0.018672	4.71E-14	0.576059	9	22164309	14306	0.5757	0.4243
	rs2249850	G	A	0.0959	-0.0256118	0.0182	0.0208183	1.42E-07	0.22992	10	104512006	14306	0.6342	0.3658
	rs28517654	C	T	-0.1356	-0.00916789	0.0205	0.0204695	3.86E-11	0.587581	4	55993468	14306	0.7568	0.2432
	rs55667387	C	T	0.0944	-0.0236175	0.0185	0.0191601	3.50E-07	0.221622	3	29771230	14306	0.6619	0.3381
	rs57439315	T	G	-0.101	0.0129113	0.0181	0.0201893	2.63E-08	0.51078	2	11733488	14306	0.6221	0.3779
	rs6546324	C	A	-0.125	-0.0118189	0.019	0.0202139	4.31E-11	0.55385	2	67856490	14306	0.3079	0.3079
	rs6908034	A	G	0.1261	0.0412476	0.024	0.0246859	1.41E-07	0.0894417	6	19773930	14306	0.8427	0.1573
	rs7455028	T	C	0.1247	0.0151189	0.0184	0.0206548	1.13E-11	0.46969	6	152034386	14306	0.6572	0.3428
	rs749749	C	T	0.0923	-0.00234828	0.0175	0.0185034	1.30E-07	0.93555	15	40350131	14306	0.5211	0.4789
	rs9485296	G	A	0.1136	0.00467919	0.022	0.0248621	2.39E-07	0.883046	6	148871389	14306	0.8022	0.1978
family Prevotellaceae	rs10757277	G	A	-0.1013	-0.00150428	0.0178	0.0118415	1.25E-08	0.913381	9	22124450	14306	0.5797	0.4203
	rs10917151	A	G	0.1685	0.0200452	0.0232	0.0153459	4.03E-13	0.230674	1	22422721	14306	0.8294	0.1706
	rs11031005	C	T	-0.2379	-0.00998371	0.0239	0.0170099	2.63E-23	0.622194	11	30226356	14306	0.8324	0.1676
	rs1566646	T	G	-0.1116	-0.0166353	0.0225	0.0136172	6.80E-07	0.211209	12	115117029	14306	0.8069	0.1931
	rs17694933	A	G	0.1331	0.0255571	0.0177	0.0117678	4.71E-14	0.0301217	9	22164309	14306	0.5757	0.4243
	rs2249850	G	A	0.0959	0.00905136	0.0182	0.0132072	1.42E-07	0.473597	10	104512006	14306	0.6342	0.3658
	rs28517654	C	T	-0.1356	0.00939372	0.0205	0.0130623	3.86E-11	0.528685	4	55993468	14306	0.7568	0.2432
	rs55667387	C	T	0.0944	0.0182268	0.0185	0.0121006	3.50E-07	0.131387	3	29771230	14306	0.6619	0.3381
	rs57439315	T	G	-0.101	0.0126486	0.0181	0.0124004	2.63E-08	0.294159	2	11733488	14306	0.6221	0.3779
	rs6546324	C	A	-0.125	-0.00539537	0.019	0.0128594	4.31E-11	0.721151	2	67856490	14306	0.3079	0.3079
	rs6908034	A	G	0.1261	-0.0140684	0.024	0.0157365	1.41E-07	0.352883	6	19773930	14306	0.8427	0.1573
	rs7455028	T	C	0.1247	-0.0038307	0.0184	0.0130704	1.13E-11	0.738115	6	152034386	14306	0.6572	0.3428
	rs749749	C	T	0.0923	-0.0118763	0.0175	0.0117262	1.30E-07	0.296601	15	40350131	14306	0.5211	0.4789
	rs9485296	G	A	0.1136	0.00210642	0.022	0.0156341	2.39E-07	0.915258	6	148871389	14306	0.8022	0.1978
genus Anaerotruncus	rs10757277	G	A	-0.1013	0.0062971	0.0178	0.0111895	1.25E-08	0.559226	9	22124450	14306	0.5797	0.4203
	rs10917151	A	G	0.1685	-0.0057443	0.0232	0.0146692	4.03E-13	0.705913	1	22422721	14306	0.8294	0.1706
	rs11031005	C	T	-0.2379	0.0362199	0.0239	0.0158934	2.63E-23	0.0367561	11	30226356	14306	0.8324	0.1676
	rs1566646	T	G	-0.1116	0.0181266	0.0225	0.0129015	6.80E-07	0.157786	12	115117029	14306	0.8069	0.1931
	rs17694933	A	G	0.1331	0.00958808	0.0177	0.0111416	4.71E-14	0.381321	9	22164309	14306	0.5757	0.4243
	rs2249850	G	A	0.0959	0.0180339	0.0182	0.0124419	1.42E-07	0.126735	10	104512006	14306	0.6342	0.3658
	rs28517654	C	T	-0.1356	0.022274	0.0205	0.0123108	3.86E-11	0.0879772	4	55993468	14306	0.7568	0.2432
	rs55667387	C	T	0.0944	0.0235393	0.0185	0.0114446	3.50E-07	0.0412962	3	29771230	14306	0.6619	0.3381
	rs57439315	T	G	-0.101	0.00168069	0.0181	0.0117454	2.63E-08	0.878437	2	11733488	14306	0.6221	0.3779
	rs6546324	C	A	-0.125	0.00807998	0.019	0.0121176	4.31E-11	0.50012	2	67856490	14306	0.3079	0.3079
	rs6908034	A	G	0.1261	0.00294477	0.024	0.0148849	1.41E-07	0.822219	6	19773930	14306	0.8427	0.1573
	rs7455028	T	C	0.1247	-0.00186664	0.0184	0.0123385	1.13E-11	0.805471	6	152034386	14306	0.6572	0.3428
	rs749749	C	T	0.0923	-0.00186725	0.0175	0.0110747	1.30E-07	0.840995	15	40350131	14306	0.5211	0.4789
	rs9485296	G	A	0.1136	0.005632	0.022	0.0148106	2.39E-07	0.723196	6	148871389	14306	0.8022	0.1978
genus Eubacterium ruminantium group	rs10757277	G	A	-0.1013	0.0217469	0.0178	0.0165796	1.25E-08	0.191583	9	22124450	14306	0.5797	0.4203
	rs10917151	A	G	0.1685	0.000561442	0.0232	0.0213155	4.03E-13	0.955673	1	22422721	14306	0.8294	0.1706
	rs11031005	C	T	-0.2379	-0.0159124	0.0239	0.0235711	2.63E-23	0.56521	11	30226356	14306	0.8324	0.1676
	rs1566646	T	G	-0.1116	0.00534898	0.0225	0.0190797	6.80E-07	0.729266	12	115117029	14306	0.8069	0.1931
	rs17694933	A	G	0.1331	-0.0136524	0.0177	0.0163294	4.71E-14	0.407347	9	22164309	14306	0.5757	0.4243
	rs2249850	G	A	0.0959	0.00481792	0.0182	0.0183941	1.42E-07	0.882579	10	104512006	14306	0.6342	0.3658
	rs28517654	C	T	-0.1356	-0.00439833	0.0205	0.0180536	3.86E-11	0.769276	4	55993468	14306	0.7568	0.2432
	rs55667387	C	T	0.0944	0.0203477	0.0185	0.0167463	3.50E-07	0.226667	3	29771230	14306	0.6619	0.3381
	rs57439315	T	G	-0.101	-0.0155865	0.0181	0.0173967	2.63E-08	0.365529	2	11733488	14306	0.6221	0.3779
	rs6546324	C	A	-0.125	0.0172482	0.019	0.0178178	4.31E-11	0.325994	2	67856490	14306	0.3079	0.3079
	rs6908034	A	G	0.1261	0.0447996	0.024	0.0218105	1.41E-07	0.0295931	6	19773930	14306	0.8427	0.1573
	rs7455028	T	C	0.1247	0.0105339	0.0184	0.0181947	1.13E-11	0.612812	6	152034386	14306	0.6572	0.3428
	rs749749	C	T	0.0923	0.0320246	0.0175	0.0162459	1.30E-07	0.0564703	15	40350131	14306	0.5211	0.4789
	rs9485296	G	A	0.1136	0.0006334	0.022	0.0216282	2.39E-07	0.989391	6	148871389	14306	0.8022	0.1978
genus Olsenella	rs10757277	G	A	-0.1013	0.00391408	0.0178	0.0232272	1.25E-08	0.879558	9	22124450	14306	0.5797	0.4203
	rs10917151	A	G	0.1685	0.0460189	0.0232	0.0308274	4.03E-13	0.157286	1	22422721	14306	0.8294	0.1706
	rs11031005	C	T	-0.2379	-0.000114463	0.0239	0.0326216	2.63E-23	0.960869	11	30226356	14306	0.8324	0.1676
	rs1566646	T	G	-0.1116	-0.00663529	0.0225	0.0269487	6.80E-07	0.823094	12	115117029	14306	0.8069	0.1931
	rs17694933	A	G	0.1331	0.0254745	0.0177	0.0233558	4.71E-14	0.281043	9	22164309	14306	0.5757	0.4243
	rs2249850	G	A	0.0959	0.0069437	0.0182	0.0258574	1.42E-07	0.759871	10	104512006	14306	0.6342	0.3658
	rs28517654	C	T	-0.1356	0.0217307	0.0205	0.025574	3.86E-11	0.387479	4	55993468	14306	0.7568	0.2432
	rs55667387	C	T	0.0944	-0.0109269	0.0185	0.0239667	3.50E-07	0.653087	3	29771230	14306	0.6619	0.3381
	rs57439315	T	G	-0.101	0.00867091	0.0181	0.0245059	2.63E-08	0.700882	2	11733488	14306	0.6221	0.3779
	rs6546324	C	A	-0.125	-0.0226547	0.019	0.0253941	4.31E-11	0.375216	2	67856490	14306	0.3079	0.3079
	rs6908034	A	G	0.1261	-0.0198553	0.024	0.0313995	1.41E-07	0.527772	6	19773930	14306	0.8427	0.1573
	rs7455028	T	C	0.1247	0.0495017	0.0184	0.0256337	1.13E-11	0.0661592	6	152034386	14306	0.6572	0.3428
	rs749749	C	T	0.0923	0.045876	0.0175	0.0232075	1.30E-07	0.0454088	15	40350131	14306	0.5211	0.4789
	rs9485296	G	A	0.1136	0.032149	0.022	0.0311931	2.39E-07	0.305944	6	148871389	14306	0.8022	0.1978
genus Oscillospira	rs10757277	G	A	-0.1013	-0.00206668	0.0178	0.0143197	1.25E-08	0.89281	9	22124450	14306	0.5797	0.4203
	rs10917151	A	G	0.1685	-0.00354135	0.0232	0.0188343	4.03E-13	0.90401	1	22422721	14306	0.8294	0.1706
	rs11031005	C	T	-0.2379	0.000524851	0.0239	0.0202183	2.63E-23	0.983183	11	30226356	14306	0.8324	0.1676
	rs1566646	T	G	-0.1116	-0.0139263	0.0225	0.0165516	6.80E-07	0.392642	12	115117029	14306	0.8069	0.1931
	rs17694933	A	G	0.1331	0.0168688	0.0177	0.0142011	4.71E-14	0.220386	9	22164309	14306	0.5757	0.4243
	rs2249850	G	A	0.0959	0.0278305	0.0182	0.0158921	1.42E-07	0.0915841	10	104512006	14306	0.6342	0.3658
	rs28517654	C	T	-0.1356	-0.0258818	0.0205	0.0156439	3.86E-11	0.0923422	4	55993468	14306	0.7568	0.2432
	rs55667387	C	T	0.0944	-0.0169871	0.0185	0.0145552	3.50E-07	0.239977	3	29771230	14306	0.6619	0.3381
	rs57439315	T	G	-0.101	-0.0118583	0.0181	0.0150339	2.63E-08	0.432276	2	11733488	14306	0.6221	0.3779
	rs6546324	C	A	-0.125	-0.00572575	0.019	0.0154039	4.31E-11	0.681499	2	67856490	14306	0.3079	0.3079
	rs6908034	A	G	0.1261	0.00908148	0.024	0.0189902	1.41E-07	0.640091	6	19773930	14306	0.8427	0.1573
	rs7455028	T	C	0.1247	-0.0267358	0.0184	0.0157558	1.13E-11	0.0808407	6	152034386	14306	0.6572	0.3428
	rs749749	C	T	0.0923	0.00800675	0.0175	0.014101	1.30E-07	0.600846	15	40350131	14306	0.5211	0.4789
	rs9485296	G	A	0.1136	-0.0104155	0.022	0.0187962	2.39E-07	0.544555	6	148871389	14306	0.8022	0.1978
order Bacillales	rs10757277	G	A	-0.1013	0.00354952	0.0178	0.0257322	1.25E-08	0.86746	9	22124450	14306	0.5797	0.4203
	rs10917151	A	G	0.1685	0.0147927	0.0232	0.0337789	4.03E-13	0.615306	1	22422721	14306	0.8294	0.1706
	rs11031005	C	T	-0.2379	-0.00311658	0.0239	0.0357573	2.63E-23	0.880654	11	30226356	14306	0.8324	0.1676
	rs1566646	T	G	-0.1116	0.0609855	0.0225	0.0298703	6.80E-07	0.0431301	12	115117029	14306	0.8069	0.1931
	rs17694933	A	G	0.1331	-0.000649089	0.0177	0.0251608	4.71E-14	0.97821	9	22164309	14306	0.5757	0.4243
	rs2249850	G	A	0.0959	-0.0381786	0.0182	0.028387	1.42E-07	0.153901	10	104512006	14306	0.6342	0.3658
	rs28517654	C	T	-0.1356	-0.00451679	0.0205	0.0276167	3.86E-11	0.847784	4	55993468	14306	0.7568	0.2432
	rs55667387	C	T	0.0944	0.0285453	0.0185	0.0257884	3.50E-07	0.271473	3	29771230	14306	0.6619	0.3381
	rs57439315	T	G	-0.101	0.0381852	0.0181	0.0270304	2.63E-08	0.146527	2	11733488	14306	0.6221	0.3779
	rs6546324	C	A	-0.125	-0.0025676	0.019	0.0272447	4.31E-11	0.915477	2	67856490	14306	0.3079	0.3079
	rs6908034	A	G	0.1261	0.0129537	0.024	0.0337124	1.41E-07	0.570964	6	19773930	14306	0.8427	0.1573
	rs7455028	T	C	0.1247	0.0208431	0.0184	0.0281268	1.13E-11	0.488415	6	152034386	14306	0.6572	0.3428
	rs749749	C	T	0.0923	0.011005	0.0175	0.0250148	1.30E-07	0.666897	15	40350131	14306	0.5211	0.4789
	rs9485296	G	A	0.1136	0.0326104	0.022	0.0331536	2.39E-07	0.299057	6	148871389	14306	0.8022	0.1978
